# Build fair machine learning models to predict adverse outcomes for heart failure patients with preserved ejection fraction and with reduced ejection fraction

**DOI:** 10.1093/jamiaopen/ooag136

**Published:** 2026-07-10

**Authors:** YaYun Yeh, YaoAn Lee, Yu Huang, Wenxi Huang, Stephen E Kimmel, Carl Yang, Zhe Jiang, Tingsong Xiao, Yi Guo, Jiang Bian, Jingchuan Guo

**Affiliations:** Department of Pharmacy Practice, Purdue University College of Pharmacy, Indianapolis, IN 46202, United States; Regenstrief Institute, Indianapolis, IN 46202, United States; Regenstrief Institute, Indianapolis, IN 46202, United States; Biostatistics and Health Data Science, Indiana University, Indianapolis, IN 46202, United States; Pharmaceutical Outcomes & Policy, University of Florida, Gainesville, FL 32611, United States; Department of Epidemiology, University of Florida, Gainesville, FL 32610, United States; Data and Information Systems Research Lab, Emory University, Atlanta, GA 30322, United States; Department of Computer & Information Science & Engineering, University of Florida, Gainesville, FL 32611, United States; Department of Computer & Information Science & Engineering, University of Florida, Gainesville, FL 32611, United States; Department of Health Outcomes and Biomedical Informatics, University of Florida, Gainesville, FL 32611, United States; Regenstrief Institute, Indianapolis, IN 46202, United States; Biostatistics and Health Data Science, Indiana University, Indianapolis, IN 46202, United States; Department of Pharmacy Practice, Purdue University College of Pharmacy, Indianapolis, IN 46202, United States; Regenstrief Institute, Indianapolis, IN 46202, United States

**Keywords:** heart failure with preserved ejection fraction (HFpEF), heart failure with reduced ejection fraction (HFrEF), machine learning (ML), prediction, fairness, social determinants of health

## Abstract

**Objectives:**

Develop and validate subtype-specific, fairness-aware machine learning (ML) models that integrate clinical and social determinants of health (SDoH) information to predict 6-month readmission or mortality after hospitalization among patients with HFpEF or HFrEF, and to evaluate the incremental contribution of SDoH, model explainability, and subgroup error disparities across demographic groups.

**Materials and Methods:**

We used University of Florida Health electronic health record (EHR) data (2016-2022) to identify adult heart failure (HF) hospitalizations and followed patients for 6 months for a composite outcome of readmission or mortality. Features included clinical characteristics, contextual SDoH (eg, neighborhood deprivation), and individual SDoH extracted from clinical notes via natural language processing (NLP). Logistic regression and XGBoost models were trained with random oversampling. Performance metrics included the C statistic, F1-score, and recall. Fairness was evaluated using false negative rate (FNR) parity across sex, race/ethnicity, and age band, and mitigation methods were applied (eg, Disparate Impact Remover, Adversarial Debiasing, and Calibrated Equalized Odds).

**Results:**

Adding SDoH improved the C statistic for logistic regression in HFpEF (0.603 vs 0.586) and HFrEF (0.641 vs 0.637). SHapley Additive exPlanations (SHAP) highlighted sodium, financial constraint level, and emergency department visit count in HFpEF, and utilization measures and financial constraint level in HFrEF. FNR ratios indicated race/ethnicity disparities; HFpEF FNRBlack/FNRWhite was 0.7834 (0.8728 after Disparate Impact Remover), and HFrEF FNRHispanic/FNRWhite was 1.2217 (0.9880 after Adversarial Debiasing).

**Discussion:**

SDoH integration and mitigation can modestly improve performance while reducing subgroup error disparities.

**Conclusion:**

Subtype-specific, fairness-aware ML models for HFpEF and HFrEF provided interpretable 6-month risk stratification and enabled subgroup fairness assessment. Integrating clinical and SDoH information added modest discrimination gains while strengthening interpretation and fairness assessment. These findings support further validation of explainable, equity-aware HF prediction models.

## Introduction

Heart failure (HF) is a leading cause of hospitalization and mortality worldwide, and it is classified into heart failure with preserved ejection fraction (HFpEF) and heart failure with reduced ejection fraction (HFrEF), each characterized by distinct pathophysiology, clinical features, and treatment approaches. HF affects more than 64 million individuals worldwide.^1^ In the United States, approximately 33.4% of HF patients have HFpEF, while 66.6% have HFrEF.^2^ Worsening HF, marked by symptomatic and clinical decline, is observed in nearly 1 in 6 HFrEF patients within 18 months of diagnosis.^3^ For HFpEF, limited treatment options and heterogeneous pathophysiology complicate clinical decision-making and risk stratification. Therefore, accurate prediction of adverse outcomes in HF is essential for guiding treatment strategies, reducing hospital readmissions, and improving quality of life. Previous studies have identified several key predictive factors for HF-related adverse events. For example, elevated NT-proBNP levels, prior HF hospitalizations, diabetes mellitus, and older age were found to be strong independent predictors of all-cause mortality and hospitalization in patients with HFpEF.^4^ In patients with HFrEF, risk factors for adverse outcomes include advanced age, renal dysfunction, anemia, and a history of myocardial infarction.^5^ Other studies have identified demographic and clinical characteristics—including older age, female sex, and polypharmacy—as relevant predictors. A post hoc analysis of the TOPCAT trial demonstrated that hyper-polypharmacy was associated with an increased risk of hospitalization and serious adverse events in patients with HFpEF.^6^ In addition, recent evidence points to contextual-level social determinants of health (SDoH), including housing instability, healthcare access, and environmental exposures, as contributing factors. Housing instability has been associated with increased emergency department use and delayed access to medical care, factors which may adversely affect chronic disease management such as in heart failure.^7^ Healthcare utilization metrics such as frequent emergency department visits and hospital admissions have also been associated with worse outcomes. Patients with frequent emergency department (ED) visits for acute HF accounted for over 55% of all HF-related hospitalizations, indicating a higher risk of adverse events.^8^

Significant disparities have been documented in both HFpEF and HFrEF and their clinical outcomes. For instance, Black patients are nearly 2.5 times more likely to be hospitalized for HF compared to White patients.^9^ These disparities underscore the importance of addressing SDoH factors, including both contextual and individual SDoH, in understanding and managing HF. Contextual-level SDoH refer to factors measured from an individual’s surroundings, encompassing both social and physical environments, such as neighborhood characteristics, healthcare quality, and the built environment. Individual-level SDoH refer to personal social and economic conditions that influence health, such as income, education, employment status, housing stability, and access to transportation. Studies have shown that both contextual and individual-level SDoH contribute to disparities in HFpEF and HFrEF patient outcomes.^9^

Despite recognizing these predictors, integrating machine learning (ML)-based prediction models into clinical HF care remains challenging due to issues such as data overload, limited clinician capacity, and underutilization of social risk information. Few studies have incorporated both clinical and contextual-level SDoH into HF risk prediction models, which is particularly concerning given HF’s high burden, heterogeneity, and known disparities in outcomes across racial-ethnic and socioeconomic groups. A personalized and fair approach for risk management is urgently needed.

Algorithmic fairness is an increasingly important consideration in clinical ML because prediction models trained on real-world healthcare data may reproduce or amplify existing disparities in access to care, diagnosis, treatment, and outcome ascertainment. Prior work has shown that healthcare algorithms can exhibit racial bias when proxy outcomes, such as healthcare cost or utilization, incompletely represent underlying clinical need.^10^ Therefore, fairness-aware clinical prediction requires not only assessment of overall discrimination and calibration, but also evaluation of subgroup-specific errors across protected attributes.^11^ In this study, we adopted equality of opportunity as the guiding fairness framework, with emphasis on whether patients who later experienced readmission or death were identified at comparable rates across demographic subgroups.^12^ This criterion is clinically relevant for heart failure risk prediction because false negative predictions may delay intensified monitoring, follow-up care, or social support for patients at elevated risk. Therefore, we evaluated false negative rate parity as the primary subgroup fairness metric.

In this study, leveraging real-world data from the University of Florida Health EHR, we developed and validated subtype-specific, fairness-aware ML models to predict 6-month readmission or mortality among patients with HFpEF and HFrEF. We evaluated the incremental contribution of individual- and contextual-level SDoH, assessed model explainability using SHAP, explored feature relationships using CPC-based causal structure learning, and examined subgroup error disparities using FNR parity.

## Methods

### Study design and population

We conducted a retrospective cohort study using the UF Health Integrated Data Repository EHRs from 2016 to 2022. The cohort included adult patients diagnosed with HFpEF or HFrEF. Identification of HFpEF and HFrEF was based on validated algorithms with international classification of diseases (ICD) diagnosis codes^13^ (Table S1). For patients with multiple eligible HF hospitalizations during the study period, we used the first recorded eligible inpatient HF encounter as the index hospitalization. Each patient contributed only 1 index hospitalization to the analytic cohort. We defined the index date as the admission date of this index hospitalization and followed patients for subsequent readmission or mortality. The workflow for cohort identification and ML model development is shown in Figure S1.

### Study outcome

The study outcome was the composite of HF readmission or death within 6 months following the index date. Readmission was identified by a subsequent inpatient encounter with a primary diagnosis of HF occurring after discharge from the index hospitalization and within the 6-month follow-up period. Additional admissions after the first qualifying readmission were not counted as separate observations because the outcome was defined as a binary composite event. Mortality was identified using the date of death recorded in the UF Health EHR, based on structured death date fields and discharge disposition status. Patients were censored at 6 months post-index or at the time of death, whichever occurred first.

### Predictor candidates

We collected a comprehensive set of demographic, clinical, and SDoH (contextual- and individual-level) variables at baseline to develop ML models for predicting 6-month HF readmission and death in HFpEF and HFrEF, respectively (Tables S2 and S3). The variables were selected based on clinical expertise and prior literature.^14^ Clinical variables were identified from structured EHR data using ICD diagnosis codes for comorbidities and RxNorm codes for medications. Individual-level SDoH variables were extracted from unstructured clinical notes using an NLP-based SDoH extraction approach^15^ across 9 domains: education, occupation, financial constraint, living condition, living supply, marital status, smoking, alcohol use, and drug use. Domain-level NLP validation metrics were not available in the current analytic dataset; therefore, these variables were interpreted as EHR-derived SDoH indicators rather than manually adjudicated SDoH measures. Contextual-level SDoH were derived from geospatially linked, community-level datasets. All candidate predictor variables were collected from the pre-index baseline period and the index hospitalization, ensuring that only information available up to discharge was used as predictors. Contextual-level SDoH measures were linked to patients using county FIPS codes and represented county-level area measures. These variables were interpreted as contextual proxies for patients’ social and environmental conditions rather than individual-level exposures. The contextual SDoH measures were based on a 2019 county-level SDoH snapshot and treated as time-fixed baseline contextual measures. Additional data-source descriptions and representative variables are provided in Supplementary Table S3.

### Development of ML pipeline for HFpEF and HFrEF prediction models

The ML development process followed 5 main steps: data preprocessing, model training, performance evaluation, explanation, and fairness assessment. Two supervised ML algorithms, logistic regression (LRC) and XGBoost, were developed independently for 2 datasets: the HFpEF and HFrEF.

#### Step 1. Preprocessing

Missing values in continuous variables were imputed using median imputation, and mode imputation was applied for categorical variables. Features with high missingness or insufficient variability were excluded to improve robustness and reduce overfitting. High missingness was defined as more than 50% missing values. For contextual-level SDoH variables, variables with more than 50% missingness were removed. For retained continuous contextual SDoH variables, remaining missing values were imputed using the median, and variables with zero variance after imputation were excluded. Categorical contextual SDoH variables were 1-hot encoded. After preprocessing, the final modeling-ready contextual SDoH feature set included 968 analytic predictors. The FIPS code variable was used only for linkage or descriptive purposes and was not treated as a model predictor.

#### Step 2. ML modeling

The dataset was split randomly into training (70%), validation (10%), and testing (20%) sets. Random oversampling was used to address class imbalance within the training set. Model development and reporting were guided by established prediction-model reporting principles.^16^ We selected logistic regression and XGBoost as complementary modeling approaches. LRC was included as a transparent and clinically interpretable baseline model that allows direct comparison of feature sets and provides relatively stable estimates in moderate-sized clinical cohorts. XGBoost was included because gradient-boosted tree models can capture nonlinear relationships and higher-order feature interactions that are common in EHR data, while often performing well with mixed variable types and sparse predictors.^17^ We limited the primary comparison to these 2 commonly used algorithms to balance predictive flexibility, interpretability, and feasibility for fairness assessment and mitigation. Both ML models were trained using grid search cross-validation to optimize hyperparameters.

#### Step 3. Performance assessment

Model performance was assessed using multiple metrics, including the C statistic, F1-score, precision, recall, and specificity.

#### Step 4. Model explanation

SHAP values were applied for model interpretability. SHAP values helped rank the importance of both clinical and SDoH features in driving adverse outcomes in different HF subtypes. To further explore the complex interactions among features, we generated a causal graph using mixed graphical models with the Conservative PC (CPC) algorithm, to identify important causal factors contributing to the outcome. This graph highlights the relationships between SDoH, comorbidities, and clinical variables, with red edges indicating indirect pathways connecting SDoH to the outcome.

#### Step 5. Fairness assessment and bias mitigation

In evaluating algorithmic fairness, we used the equality of opportunity metric, measured by the FNR to assess fairness across subgroups defined by sex, race, and ethnicity. The FNR represents the probability of incorrectly predicting no adverse event for patients who experienced one. To mitigate potential biases, we implemented 3 fairness-enhancing methods representing different stages of the ML pipeline: pre-processing (Disparate Impact Remover), in-processing (Adversarial Debiasing), and post-processing (Calibrated Equalized Odds). These approaches were selected because they are commonly used fairness mitigation methods and are available in established open-source fairness toolkits.^18^ Disparate Impact Remover weakens associations between features and protected attributes while preserving ranks. Adversarial Debiasing trains a predictor while discouraging group-dependent predictions. Calibrated Equalized Odds applies group-specific post-processing to reduce error-rate disparities while preserving calibration.^18^ We report the C statistic and FNR metrics before and after each mitigation method.

## Results

### Descriptive statistics of the study cohort

Our final cohort comprised 6207 eligible patients hospitalized for HFpEF or HFrEF, including 1417 patients with HFpEF and 4790 patients with HFrEF. Table 1 shows the study cohort’s demographics and clinical characteristics stratified by 3 collapsed predicted-risk groups: top 10%, 10%-50%, and bottom 50%. These groups were used to contrast the highest-risk decile with moderate- and lower-risk patients while maintaining adequate sample size for descriptive comparisons.

**Table 1.** Demographic and clinical characteristics of the study cohort stratified by the predicted risk of 6-month HF readmission/death risk deciles (a) HFpEF cohort and (b) HFrEF cohort

(a) HFpEF cohort				
Name^a^	Top 10 decile	10-50 decile	Bottom 50 decile	*P* value
Age, years	71.1 (12.4)	67.4 (12.8)	64.4 (13.6)	<.001
BMI (kg/m)	31.5 (9.3)	34.1 (11.5)	36.3 (11.0)	<.001
Weight (kg)	88.2 (27.6)	96.7 (34.2)	104.5 (33.0)	<.001
Heart rate (beats/minute)	74.8 (12.7)	77.2 (15.3)	79.4 (15.1)	.023
Systolic blood pressure (mmHg)	132.5 (25.6)	138.8 (25.0)	140.0 (23.7)	.01
Diastolic blood pressure (mmHg)	71.3 (13.0)	73.4 (14.2)	75.0 (14.8)	.094
Nitrate use	27 (27.27%)	72 (18.18%)	59 (11.92%)	<.001
Positive inotropic agents	1 (1.01%)	7 (1.77%)	11 (2.22%)	.696
Loop diuretics	45 (45.45%)	120 (30.30%)	126 (25.45%)	<.001
Non-loop diuretics	14 (14.14%)	42 (10.61%)	60 (12.12%)	.573
SGLT2 inhibitors	0 (0.00%)	1 (0.25%)	3 (0.61%)	.568
Beta-blockers	40 (40.40%)	143 (36.11%)	144 (29.09%)	.022
Calcium channel blockers	26 (26.26%)	101 (25.51%)	153 (30.91%)	.184
Non-insulin glucose-lowering drugs	7 (7.07%)	38 (9.60%)	69 (13.94%)	.045
Insulin use	34 (34.34%)	107 (27.02%)	113 (22.83%)	.041
Anticoagulants	16 (16.16%)	62 (15.66%)	78 (15.76%)	.992
Atrial fibrillation	43 (43.43%)	122 (30.81%)	151 (30.51%)	.035
Chronic obstructive pulmonary disease	59 (59.60%)	208 (52.53%)	272 (54.95%)	.428
Diabetes mellitus	59 (59.60%)	208 (52.53%)	272 (54.95%)	.428
Hypertension	83 (83.84%)	305 (77.02%)	392 (79.19%)	.317
Ischemic cardiovascular disease	70 (70.71%)	201 (50.76%)	206 (41.62%)	<.001
History of myocardial infarction	25 (25.25%)	66 (16.67%)	64 (12.93%)	.007
Anemia	58 (58.59%)	209 (52.78%)	225 (45.45%)	.017
Obesity	25 (25.25%)	135 (34.09%)	210 (42.42%)	.001
Serum creatinine (mg/dL)	2.1 (1.9)	1.8 (2.1)	1.4 (1.2)	<.001
LDL cholesterol (mg/dL)	86.5 (43.9)	90.0 (47.9)	88.7 (39.1)	.681
HDL cholesterol (mg/dL)	50.1 (22.6)	46.3 (20.3)	49.0 (17.2)	.058
Hemoglobin (g/dL)	7.2 (2.3)	7.2 (2.2)	6.9 (2.0)	.438

(b) HFrEF cohort				
Name^a^	Top 10 decile	10--50 decile	Bottom 50 decile	*P* value
Age, years	65.4 (13.5)	65.1 (13.6)	61.0 (14.5)	<.001
BMI (kg/m)	28.7 (8.1)	30.8 (8.9)	33.7 (10.0)	<.001
Weight (kg)	85.7 (24.9)	90.7 (28.9)	98.4 (31.4)	<.001
Heart rate (beats/minute)	87.2 (16.4)	82.4 (15.7)	79.6 (14.8)	<.001
Systolic blood pressure (mmHg)	121.3 (23.2)	130.1 (24.2)	140.0 (25.5)	<.001
Diastolic blood pressure (mmHg)	72.0 (14.9)	74.6 (15.0)	79.6 (15.8)	<.001
Nitrate use	93 (27.76%)	238 (17.76%)	203 (12.11%)	<.001
Positive inotropic agents	40 (11.94%)	46 (3.43%)	27 (1.61%)	<.001
Loop diuretics	154 (45.97%)	373 (27.84%)	292 (17.42%)	<.001
Non-loop diuretics	110 (32.84%)	186 (13.88%)	102 (6.09%)	<.001
SGLT2 inhibitors	8 (2.39%)	3 (0.22%)	0 (0.00%)	<.001
Beta-blockers	183 (54.63%)	457 (34.10%)	402 (23.99%)	<.001
Calcium channel blockers	81 (24.18%)	258 (19.25%)	297 (17.72%)	.021
Non-insulin glucose-lowering drugs	43 (12.84%)	133 (9.93%)	138 (8.23%)	.021
Insulin use	109 (32.54%)	273 (20.37%)	251 (14.98%)	<.001
Anticoagulants	85 (25.37%)	204 (15.22%)	159 (9.49%)	<.001
Atrial fibrillation	153 (45.67%)	379 (28.28%)	328 (19.57%)	<.001
Chronic obstructive pulmonary disease	202 (60.30%)	600 (44.78%)	526 (31.38%)	<.001
Diabetes mellitus	202 (60.30%)	600 (44.78%)	526 (31.38%)	<.001
Hypertension	285 (85.07%)	949 (70.82%)	1035 (61.75%)	<.001
Ischemic cardiovascular disease	256 (76.42%)	697 (52.01%)	619 (36.93%)	<.001
History of myocardial infarction	122 (36.42%)	273 (20.37%)	191 (11.40%)	<.001
Anemia	175 (52.24%)	519 (38.73%)	500 (29.83%)	<.001
Obesity	60 (17.91%)	322 (24.03%)	508 (30.31%)	<.001
Serum creatinine (mg/dL)	2.0 (1.9)	1.9 (2.1)	1.6 (2.0)	.001
LDL cholesterol (mg/dL)	79.9 (40.9)	84.9 (38.7)	90.1 (39.9)	.001
HDL cholesterol (mg/dL)	47.1 (20.1)	46.5 (17.5)	47.8 (17.4)	.369
Hemoglobin (g/dL)	6.7 (1.6)	7.0 (2.1)	6.8 (2.1)	.102

a All comorbidities and medication variables listed are binary indicators (1 = yes, 0 = no).

Of the cohort, the mean age was 64.0 ± 14.1 years, 43.4% were female, 53.26% were NHW, 40.12% were NHB, 26.36% were Hispanic, 3.17% were of other races and ethnicities, and 3.04% were of unknown. NHB and Hispanic patients were generally younger than NHW patients (60.81 and 61.92 years vs 66.48 years, respectively). Among the HFpEF cohort, hypertension (79.3%), COPD (54.2%), and diabetes (54.2%) were most prevalent; in the HFrEF cohort, hypertension (67.3%), ischemic cardiovascular disease (46.6%), and COPD (38.6%) were most prevalent.

### ML prediction models in HFpEF and HFrEF

**Figure 1.**
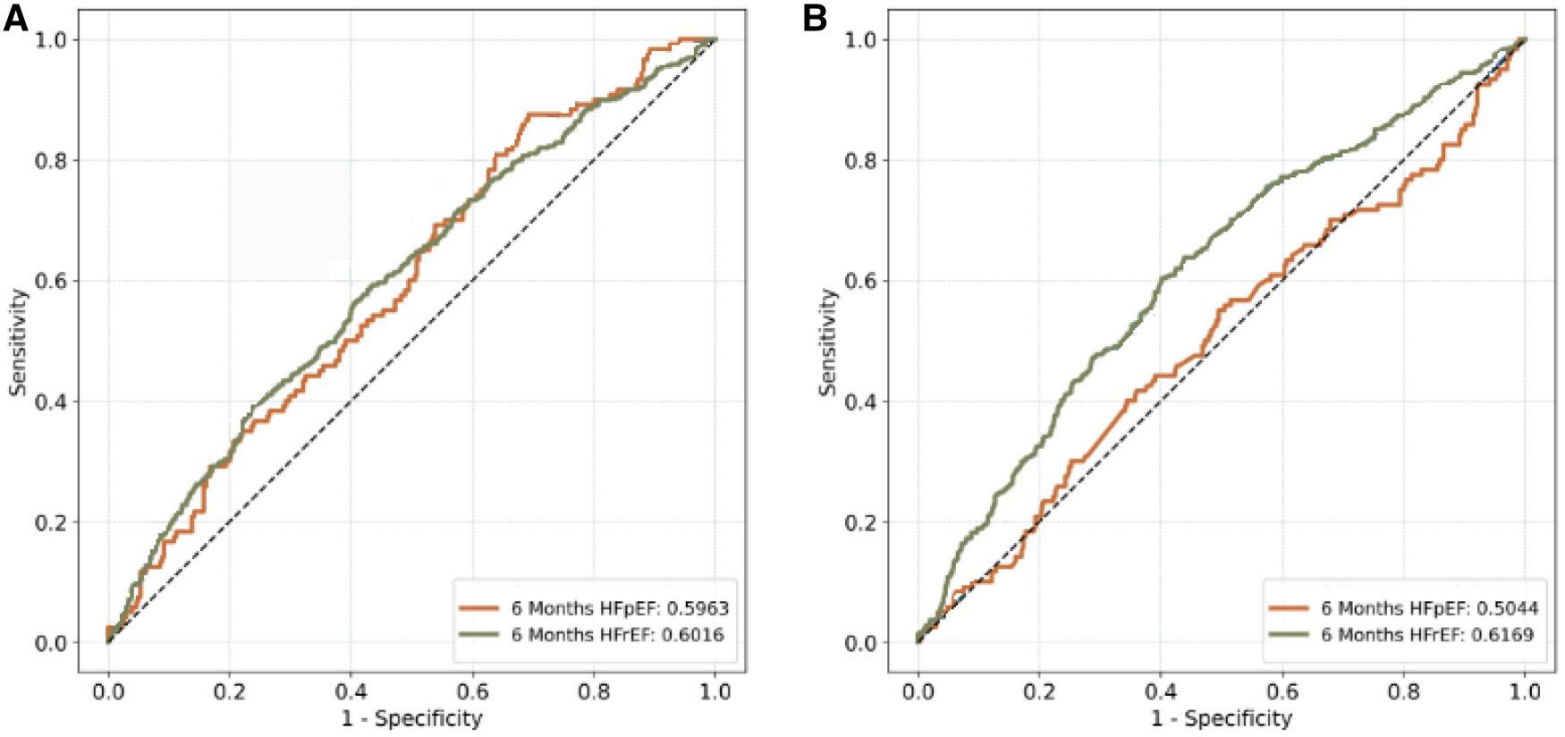
Model performance of LRC and XGBoost. Each plot shows ROC curves for models trained on HFpEF-cohort, HFrEF-cohort. (A) LRC (B) XGBoost.

We compared 3 sets of model performance: clinical only, clinical with individual SDoH and full variables (clinical plus both individual and contextual SDoH.) For the LRC model in the HFpEF cohort, the C statistic was 0.5863, 0.5805, and 0.6032 (Table S4; in HFrEF were 0.637, 0.6486 and 0.6407). Adding individual SDoH to clinical variables yielded the largest numerical gain in the HFrEF cohort, whereas adding contextual SDoH on top of clinical plus individual SDoH yielded the largest gain in the HFpEF cohort. However, the increases in the C statistic after adding SDoH were small. Because confidence intervals or formal statistical tests for differences in the C statistic were not estimated, these results should be interpreted as descriptive evidence of incremental predictive information rather than definitive evidence of statistically significant improvement in discrimination. Because LRC achieved comparable discrimination to XGBoost and offers more stable and interpretable coefficients, we used the LRC models as the primary models for SHAP-based explainability and causal discovery analyses. Figure 1 presents the receiver operating characteristic (ROC) curves illustrating the performance of the LRC and XGBoost prediction models with random oversampling.

**Figure 2.**
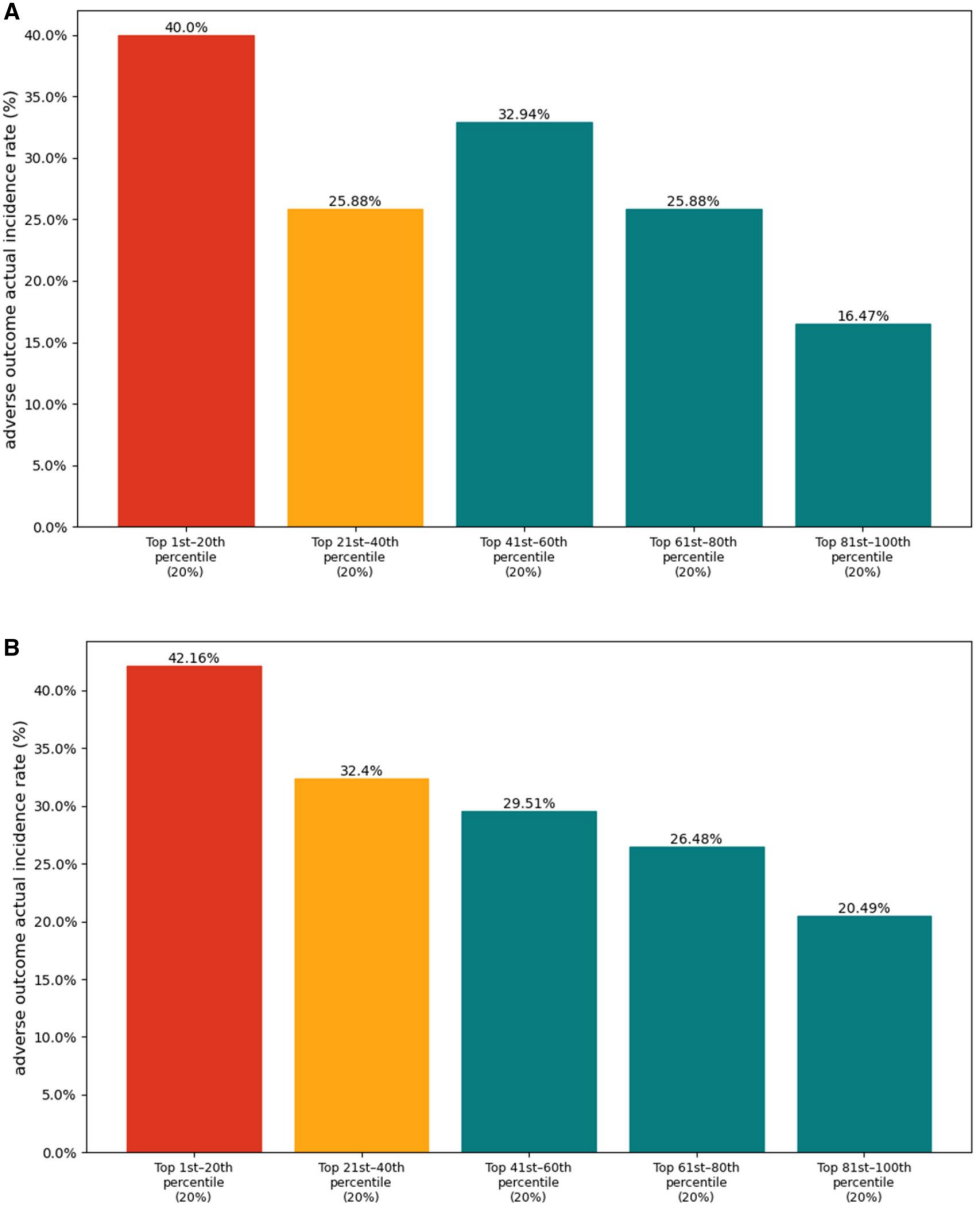
Six-month readmission/death risk by machine learning prediction risk decile using LRC model. (A) HFpEF cohort and (B) HFrEF cohort.

In the independent evaluation dataset, we stratified the predicted 6-month risk into quintiles using the ML-generated risk score to display the graded relationship between predicted risk and observed 6-month readmission or death. Quintiles were used in Figure 2 to provide a more stable visual summary of risk gradients across predicted-risk groups, demonstrating excellent calibration utility in identifying HF individuals at risk of adverse outcomes. For example, in the LRC HFpEF group, the 6-month risk of HF adverse outcomes in the top 20% of patient cohort was 40%, more than 2 times that of the bottom 20% risk group (Figure 2A). Similarly, in the XGBoost model (Figure S2), the top 20% predicted cases accounted for 41.46% of the 6-month adverse outcomes.

### Explainable AI to identify important features contributing to ML models predicting adverse outcomes in HFpEF and HFrEF

**Figure 3.**
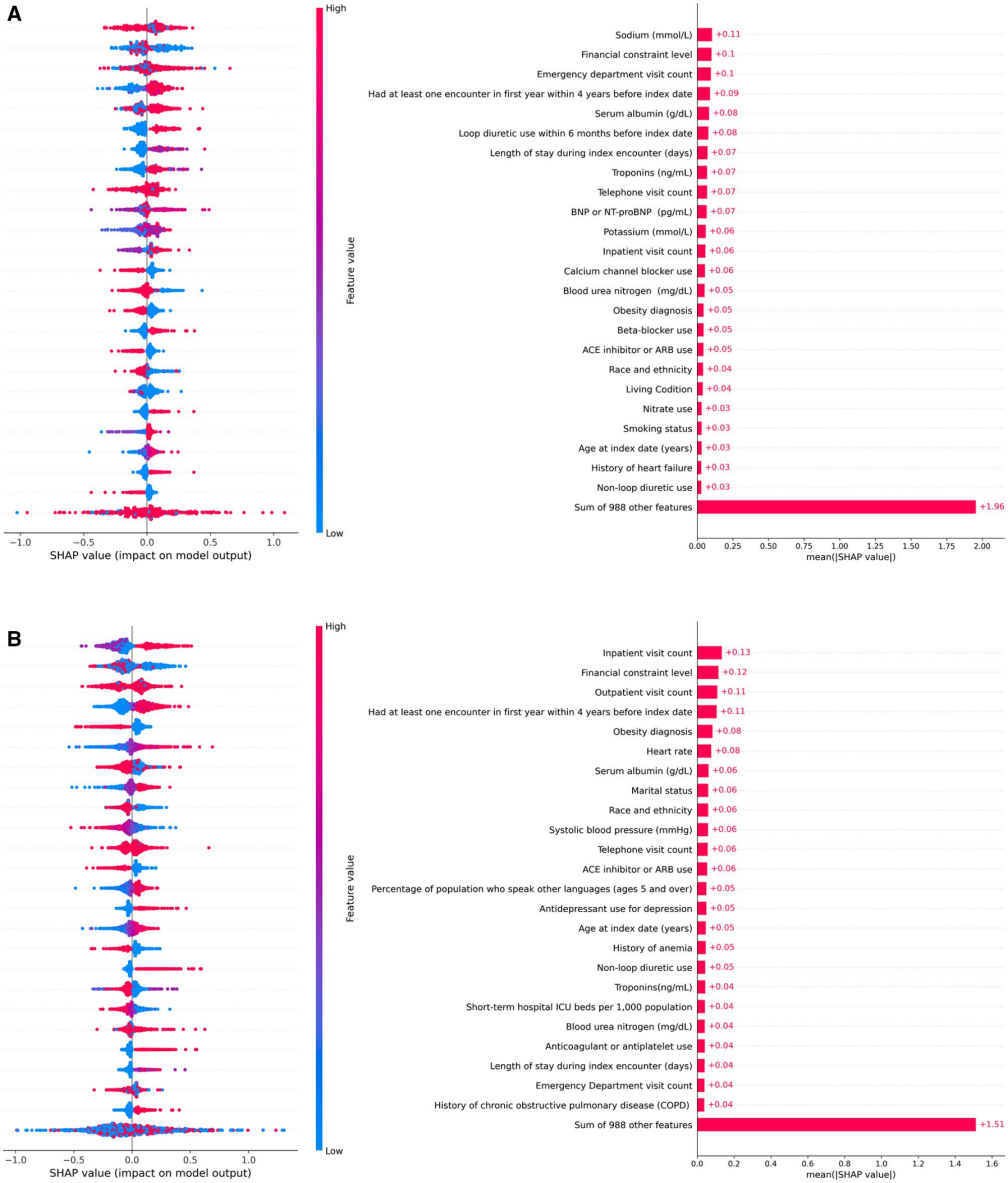
SHAP values of important predictions from the original LRC. (A) HFpEF cohort and (B) HFrEF cohort.

The 2 HF subtypes identified different sets of important features for predicting HF readmission and mortality outcomes in the LRC model (Figure 3). In HFpEF, sodium, financial constraint level, and emergency department visit count were the top 3 most important predictors, while in the HFrEF cohort, inpatient visit count, financial constraint level, and outpatient visit count were identified as the most important predictions. In contrast, the XGBoost model (Figure S3) assigned a different relative importance to these variables’ serum albumin, financial constraint level, Age at index data for HFpEF, whereas inpatient visit count, systolic blood pressure, and financial constraint level, which was identified as the most important in model’s prediction of adverse outcomes.

**Figure 4.**
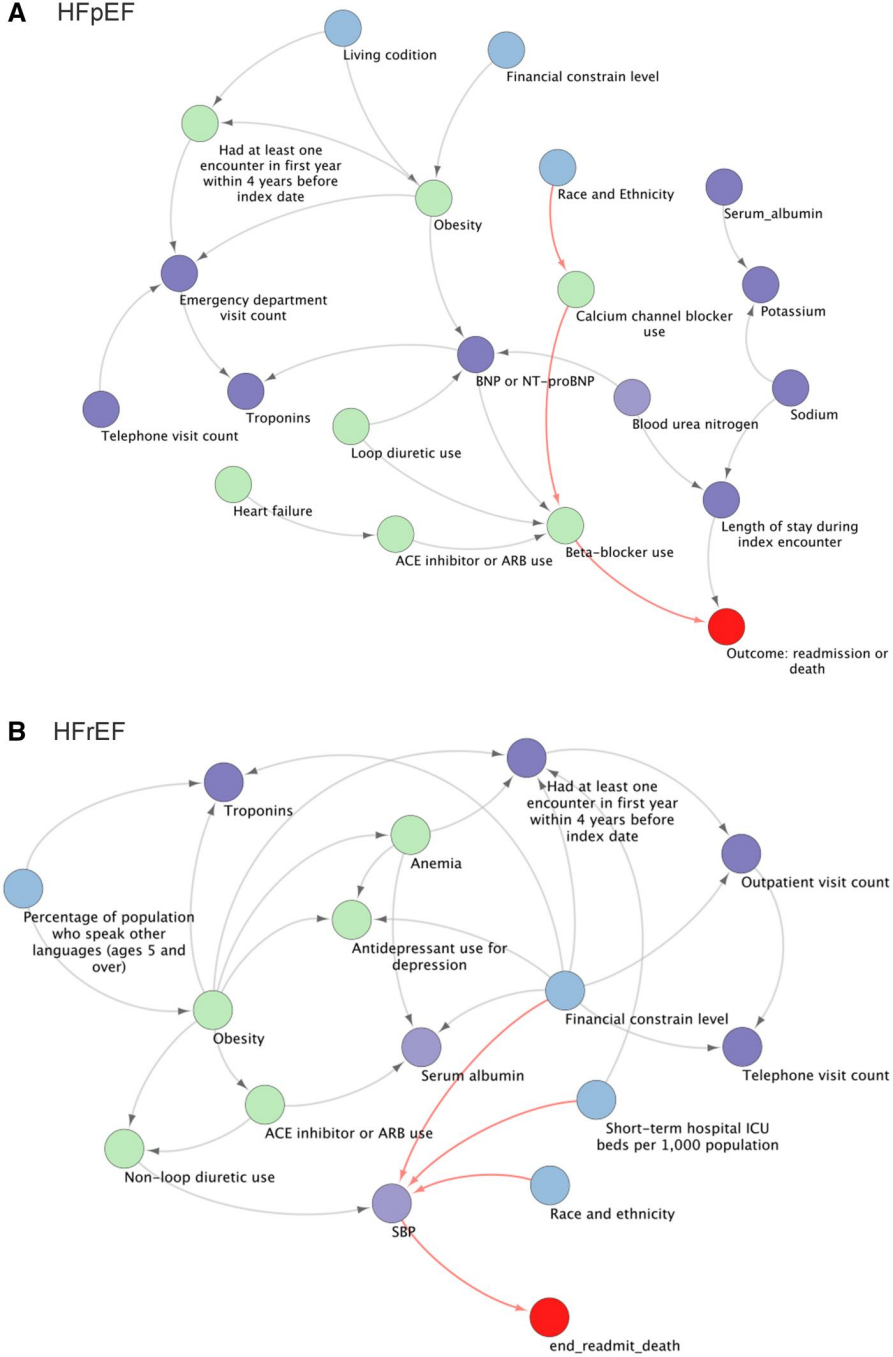
The causal discovery results on full data with LRC model from SHAP analysis. These 2 images are results from CPC models. The blue nodes present SDoH and demographics variables, the green nodes stand for comorbidities and medication variables, purple nodes stand for the clinical variables, and the red node indicates the outcome. The red edges represent the indirect relationships between SDoH and outcome. (A) HFpEF cohort and (B) HFrEF cohort.

Figure 4 shows the causal structure learning results using the CPC algorithm across the 2 different HF cohorts: (A) HFpEF and (B) HFrEF. These 2 graphs represent our exploratory analysis using causal discovery to identify potential relationships among demographic, clinical, and SDoH features. In the LRC model for the HFpEF population cohort, we identified 1 demographic variable, 9 clinical variables, 7 comorbidities, 2 individual level SDoH variables. The resulting causal graph included 20 nodes (including the outcome) and 23 edges, representing direct or indirect relationships among these variables. In the HFrEF-only cohort, we identified 1 demographic variable, 6 clinical variables, 5 comorbidities, and 1 individual-level SDoH. The resulting causal graph included 16 nodes and 28 edges. Notably, 2 indirect relationships between SDoH variables and the outcome were observed in HFpEF cohorts, while 1 indirect relationship was identified in the HFrEF cohort. Among the SDoH variables, financial constraint was found to be causally related to adverse outcomes in patients with HFpEF and HFrEF, either directly or indirectly in both the LRC and XGBoost models (Figure S4).

### Fairness assessment and mitigation

**Figure 5.**
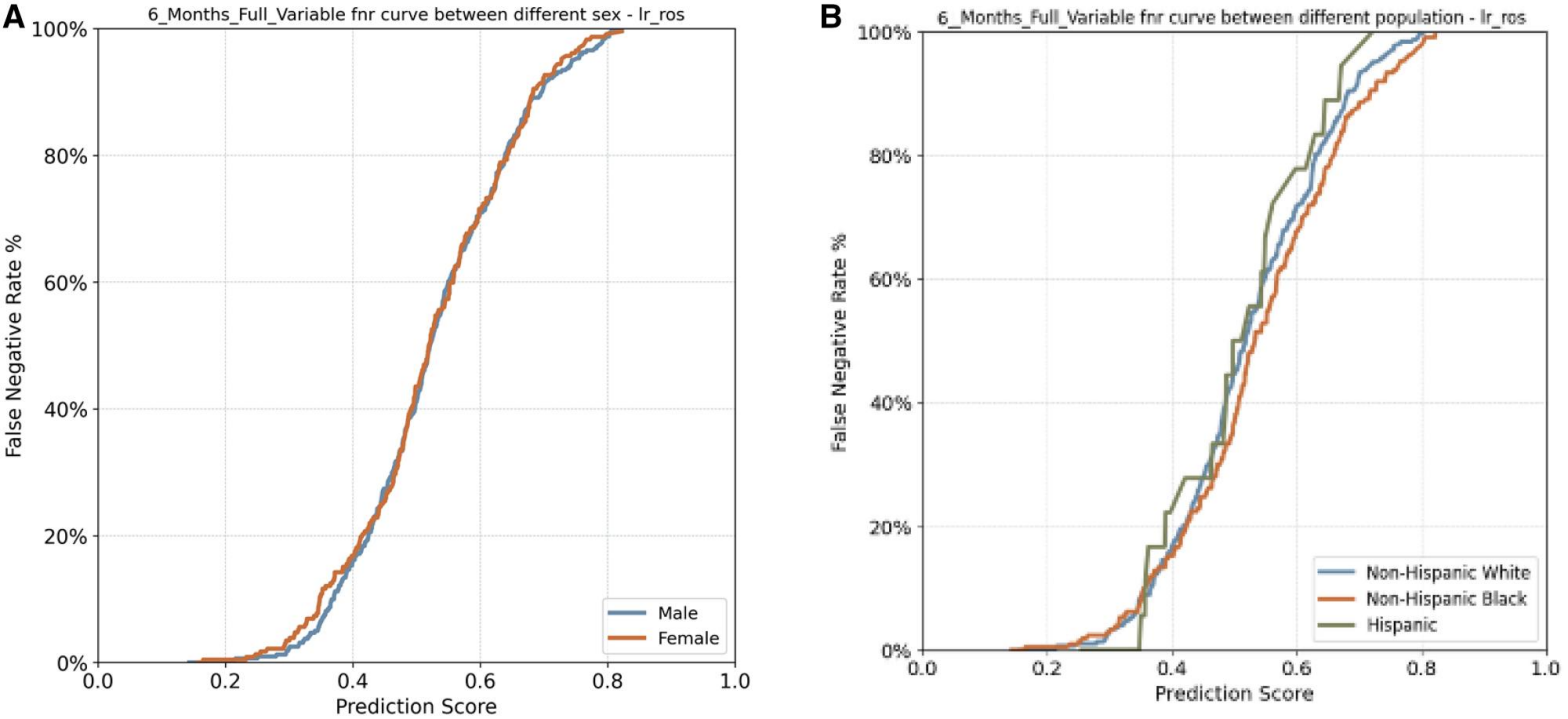
Evaluation of algorithm fairness with a focus on FNR disparity with LRC (A) by male and female and (B) by non-Hispanic white, non-Hispanic Black and Hispanic.

We assessed fairness in our ML models by evaluating FNR parity across subgroups by sex (male, female) and race/ethnicity (Hispanic, White; Black, White). An FNR ratio greater than 1 indicates under-identification in the subgroup. A detailed summary of FNR ratios across subgroups and models is provided in Table S5. The corresponding subgroup FNR curves for these groups in the LRC and XGBoost models (Figure 5), where closer overlap reflects better parity.

A notable disparity was observed in the HFpEF subtype, with a bias toward Black patients (FNR_black_/FNR_white_ = 0.7834), indicating fewer missed Black than White patients. After applying Disparate Impact Remover, the ratio improved to (FNR_black_/FNR_white_ = 0.8728). In the HFrEF subtype, the Hispanic-to-White FNR ratio was (FNR_hispanic_/FNR_white_ = 1.2217), indicating bias toward Hispanic patients, it was mitigated by Adversarial Debiasing (FNR_hispanic_/FNR_white_ = 0.9880), approching parity.

To address these biases, we applied 3 fairness-enhancing techniques: Disparate Impact Remover, Adversarial Debiasing, and Calibrated Equalized Odds, all of which improved FNR parity, though with some tradeoffs in model discrimination (the C statistic) (Figure S6). Calibration curves also demonstrated improved alignment between predicted and observed risks across demographic subgroups, indicating greater reliability of the mitigated models.

## Discussion

In this study, we developed subtype-specific ML models to predict 6-month adverse outcomes in patients with HFpEF and HFrEF, with careful attention to algorithmic fairness by applying fairness-aware mitigation techniques to enhance model explainability through SHAP values and causal inference via CPC algorithms. We found substantial differences in risk predictors and performance between the HF subtypes: the top 20% of patients identified by our models accounted for 40% of adverse events in HFpEF and 41.46% in HFrEF. However, the incremental predictive utility of SDoH should be interpreted cautiously. In this study, the primary contribution of SDoH was not a large increase in the C statistic, but rather the addition of complementary social risk information for model interpretation, risk stratification, and subgroup fairness assessment. Fairness assessments identified and effectively mitigated disparities in predictions related to sex and race/ethnicity through targeted fairness-aware methods. These results highlight the critical importance of tailored, explainable, and equitable prediction models that incorporate both clinical and social factors to improve clinical decision-making and health outcomes for diverse heart failure populations.

To our knowledge, this is among the first studies to develop HF subtype-specific prediction models of adverse outcomes among HFrEF and HFpEF patients, with careful consideration of algorithmic fairness and model explainability. We conducted separate models for HFpEF and HFrEF because these syndromes differ fundamentally in their underlying pathogenesis, risk factor profiles, disease trajectories, and therapeutic strategies. HFpEF is characterized by diastolic dysfunction, systemic inflammation, and metabolic comorbidities, whereas HFrEF involves systolic impairment, neurohormonal activation, and more established guideline directed medical therapies. A general approach would therefore obscure these subtype-specific drivers of risk. The clinical implications of our models are substantial. The model effectively identified patients at highest risk for adverse outcomes. In the HFpEF cohort, the top 20% of patients by predicted risk accounted for 40% of 6-month adverse events, whereas in the HFrEF cohort it captured 41.46%. Such subtype‐specific stratification underscores the model’s ability to pinpoint those most likely to experience readmission or death and highlights the value of tailored interventions for both HFpEF and HFrEF patients (Figure 2).

Another unique aspect of the study is that we included not only clinical factors but also individual- and contextual- level SDoH for the prediction of adverse outcomes in both HFrEF and HFpEF. Health disparities in both HFrEF and HFpEF are significant, particularly among individuals from socially and economically disadvantaged populations. For example, studies have shown that social determinants such as food insecurity, housing instability, and limited access to transportation are independently associated with higher rates of hospitalization and mortality in HF patients, and that incorporating measures of neighborhood deprivation and individual‐level socioeconomic status into risk models uncovers vulnerabilities not captured by clinical variables alone.^19^ In our study, incorporating SDoH variables slightly improved model performance compared to clinical characteristics alone. Across both cohorts, the full feature set (clinical, individual, and contextual SDoH) yielded small increases in the C statistic over clinical-only models (HFpEF, 0.5863 to 0.6032; HFrEF, 0.6370 to 0.6407). In HFrEF, the gain was driven primarily by individual-level SDoH from 0.6370 to 0.6486. In HFpEF, the C statistic increased from 0.5863 to 0.6032 when individual and contextual SDoH were added. Although these differences in the C statistic are modest, SDoH contributed complementary information, highlighting non-clinical drivers of risk and supporting more equitable identification of high-risk patients in cohorts assessments. Contextual factors were particularly predictive in HFpEF patients, whereas individual-level SDoH contributed most of the gain in HFrEF patients. These findings imply the need for subtype-specific risk models that incorporate social and environmental context.

Our prediction models offer enhanced explainability, strengthening their clinical implications. By incorporating explainable AI tools such as SHAP values and causal structure learning techniques, CPC algorithms, our models provide interpretable insights into risk prediction. Distinct key predictors were identified for HFrEF (eg, BMI, left ventricular ejection fraction, diastolic blood pressure, and inpatient visit count) and HFpEF (eg, length of stay at index encounter, BMI, diastolic blood pressure, serum albumin, and triglycerides). The causal structure learning framework additionally revealed potential causal links between SDoH (eg, percentage of Medicare-only insured individuals, percentage of American Indian and Alaska native population, and neighborhood premature death rate) and clinical variables leading to adverse outcomes in HFrEF and HFpEF, suggesting the importance of integrating social risk management into the clinical care of HF (Figure 4 and Figure S4).

Our fairness assessment revealed robust performance across racial and ethnic groups, with FNR parity metrics within acceptable statistical fairness ranges (0.80-1.25). Nevertheless, we identified subtype-specific disparities between Black and White patients in HFpEF (FNR_Black_/FNR_White_ = 0.7834), and Hispanic and White patients in HFrEF (FNR_Hispanic_/FNR_White_ = 1.2217). These findings highlight that fairness challenges are context-dependent and require tailored mitigation strategies. We addressed these disparities using 3 fairness-aware ML techniques: Disparate Impact Remover, Adversarial Debiasing, and Calibrated Equalized Odds. Each method effectively reduced subtype-specific disparities, though slightly compromising the C statistic performance, reflecting inherent tradeoffs between fairness and accuracy. Further validation in larger, multi-institutional cohorts is needed to refine and generalize these fairness strategies.

Our study is subject to several limitations. First, we did not formally test whether the observed differences in the C statistic between clinical-only and SDoH-augmented models were statistically significant. Future work should estimate confidence intervals for differences in model performance, for example using bootstrap resampling or paired tests for correlated the C statistics. Second, contextual-level SDoH measures were available only at the county level and were treated as time-fixed proxies. Because the analytic contextual SDoH variables were based on a 2019 county-level snapshot, they were not dynamically updated according to each patient’s index date or during the 6-month follow-up period. This temporal mismatch may have attenuated the contribution of contextual SDoH to model discrimination and limits interpretation of these variables as contemporaneous exposures. Future studies should use more spatially granular and temporally aligned SDoH measures, such as census-tract-level or address-level variables updated close to the index hospitalization date. Cohort identification via structured EHR diagnosis codes may include misclassified or false-positive cases, especially in nonspecific HF presentations. Future studies should incorporate NLP-driven phenotype refinement for improved accuracy. Although UF Health provides diverse patient data, this single-health-system EHR cohort may differ from nationally representative HF populations in demographics, disease severity, healthcare access, and care settings. These differences may affect model performance and fairness estimates because predictor distributions, outcome ascertainment, and subgroup-specific error rates may vary across health systems. External validation in geographically diverse, multi-institutional cohorts is needed before clinical deployment. Our individual-level SDoH variables were limited to EHR-available data and were extracted from clinical notes using NLP. Domain-level validation metrics for the extracted SDoH variables were not available for this analytic cohort; therefore, misclassification of NLP-derived SDoH features may have affected feature importance, model performance, and fairness estimates. Future work should validate SDoH extraction against manual annotation, report domain-specific performance metrics, and integrate validated SDoH screening tools. Mortality ascertainment relied on deaths recorded within the UF Health EHR. Deaths occurring outside the system were likely missed, which could underestimate event rates and affect model calibration and discrimination. Additionally, potential overlaps between predictors and outcome definitions (eg, prior hospitalization predicting readmission) may affect causal inference. Sensitivity analyses excluding overlapping predictors would strengthen future studies.

## Conclusion

Subtype-specific ML models for HFpEF and HFrEF offer interpretable 6-months risk stratification for readmission or mortality and enable assessment of subgroup error disparities. Adding individual and contextual SDoH produced small changes in overall discrimination and should be interpreted cautiously. However, SDoH features contributed complementary information for model interpretation, risk stratification, and fairness assessment. These findings support further development and external validation of explainable, equity-aware HF prediction models that integrate clinical and social risk information.

## Supplementary Material

ooag136_Supplementary_Data

## Data Availability

The data underlying this article cannot be shared publicly due to privacy regulations, Institutional Review Board restrictions, and data use agreements with UF Health. The data will be shared on reasonable request to the corresponding author, subject to approval by the relevant data custodians and ethics committees.
