# Supplementary material for: Build fair machine learning models to predict adverse outcomes for heart failure patients with preserved ejection fraction and with reduced ejection fraction
Source: JAMIA Open. 2026 Jul 10;9(4):ooag136. doi: 10.1093/jamiaopen/ooag136 (PMC13353219; doi:10.1093/jamiaopen/ooag136)

**Supplemental materials**

**Table S1.** ICD Codes for HF Definitions

**Table S2.** Summary of Variables by Category

**Table S3.** Contextual-level SDoH variables inventory by data source and domain before and after preprocessing

**Table S4.** Performance metrics for XGBoost and Logistic regression

**Table S5.** Opportunity of equality measured by false negative rate by different models on various feature sets

**Figure S1.** Workflow to identify cohort and build up ML model.

**Figure S2.** 6-month readmission/death risk by machine learning prediction risk decile using XGboost model. (a.) HFpEF population (b.) HFrEF population

**Figure S3**. SHAP values of important predictions from the original XGboost. (a.) HFpEF (b.) HFrEF.

**Figure S4.** The causal discovery results on full data with XGBoost model from SHAP analysis. These three images are results from CPC models. The blue nodes present SDoH and demographics variables, the green nodes stand for comorbidities and medication variables, purple nodes stand for the clinical variables, and the red node indicates the outcome. The red edges represent the indirect relationships between SDoH and outcome. (a.) HFpEF (b.) HFrEF

**Figure S5**. Evaluation of algorithm fairness with a focus on False negative rate disparity with XGBoost. By (a.) sex, (b.) non-Hispanic white, non-Hispanic black and Hispanic

**Figure S6**. Impact of bias mitigation on Model Performance. The x-axis shows **Equality of Opportunity**, and the y-axis shows **C statistic**. Mitigation models reduced bias but also lowered C statistic (a.) **FNR Ratio: Black/White** in HFpEF (b.) **FNR Ratio: Hispanic/White** in HFrEF population.

**Table S1.** ICD Codes for Heart Failure

| Condition |  |  |
| --- | --- | --- |
| HF | 402.x1 (402.01, 402.11, 402.91), 404.x1 (404.01, 404.11, 404.91), 404.x3 (404.03, 404.13, 404.93), 428, 428.x, 398.91 | I50, I50.x, I11.0, I13.0, I13.2, I97.13, I09.81 |
| HFpEF | 428.3x | I50.3x |
| HFrEF | 428.2x | I50.2x |

**Table S2.** Summary of Variables by Category

| Category | Variables list |
| --- | --- |
| Demographics | AGE, RACE_ETHNICITY, SEX |
| Clinical | Length of stay, Mechanical ventilation, HFpEF, heart failure history, Atrial fibrillation and/or flutter , COPD, Diabetes, Hypertension, Dyslipidemia, Ischemic cardiovascular disease, History of myocardial infarction, History of Stroke/TIA, Chronic kidney disease , Dialysis , Anemia, Depression , Cardiac and vascular device, Obesity , ICD and/or CRT-D  Pacemaker and/or CRT-P, BMI, Weight, SBP, DBP, Heart rate, Female/Male Breast Cancer , Colorectal Cancer , Prostate Cancer , Lung Cancer, Endometrial Cancer, inpatient count, outpatient count, ED count |
| Medication | Sacubitril/Valsartan_baseline, SGLT2 inhibitors, Loop diuretics, non-loop diuretics, ACEIs, ARBs, Beta blockers, Calcium channel blockers, Positive inotropic agents, Nitrates, Statins, Insulin (use any type of insulin), non-insulin glucose lowering meds (any use: metformin, DPP-4, sulfonylureas, TZD, SGLT2i and GLP1a), Anticoagulants (warfarin and DOACs), Antiplatelets, Antidepressants |
| Laboratory | Hemoglobin, Lymphocyte count, BNP / NT-proBNP, Troponins/ High-sensitivity troponin, Total cholesterol, LDL, HDL, Triglycerides, eGFR, Serum creatinine, Blood urea nitrogen, Sodium, Potassium, Serum albumin |
| Individual SDoH | Education, occupation, Financial constrain, living condition, living supply, marital status, smoking, alcohol, drug use |
| Contextual SDoH | See supplementary table S3 |

**Table S3.** Contextual-level SDoH variables inventory by data source and domain before and after preprocessing

| **Data Source** | **Contextual**  **SDoH**  **variables** | **Domain** | **After preprocess** | **Additional**  **Dummy**  **Variables**  **generated** |
| --- | --- | --- | --- | --- |
| Air Quality Index (AQI) | 15 | 4. Physical infrastructure | 12 |  |
| Air Quality System (AQS) | 14 | 4. Physical infrastructure | 4 |  |
| American Community Survey (ACS) | 309 | 1. Social Context | 124 |  |
|  |  | 2. Economic context | 79 |  |
|  |  | 3. Education | 10 |  |
|  |  | 4. Physical Infrastructure | 71 |  |
|  |  | 5. Healthcare context | 24 |  |
| American Hospital Association Annual Survey (AHA) | 6 | 5. Healthcare context | 6 |  |
| Area Health Resource Files (AHRF) | 173 | 1. Social context | 3 |  |
|  |  | 2. Economic context | 1 |  |
|  |  | 4. Physical Infrastructure | 5 |  |
|  |  | 5. Healthcare context | 160 | 5 |
|  |  | 6. Geography | 1 |  |
| CDC Interactive Atlas of Heart Disease and Stroke (CDCA) | 6 | 5. Healthcare context | 6 |  |
| CDC PLACES: Local Data for Better Health (CDCP) | 18 | 5. Healthcare context | 18 |  |
| Wide-ranging Online Data for Epidemiologic Research (CDC WONDER) | 8 | 5. Healthcare context | 7 |  |
| County Health Rankings & Roadmaps Data (CHRD) | 86 | 1. Social Context | 2 |  |
|  |  | 2. Economic context | 10 |  |
|  |  | 3. Education | 2 |  |
|  |  | 4. Physical infrastructure | 15 |  |
|  |  | 5. Healthcare context | 45 |  |
| Census Bureau Small Area Health Insurance Estimates (SAHIE) | 7 | 5. Healthcare context | 7 |  |
| Census Bureau Small Area Income and Poverty Estimates (SAIPE) | 7 | 2. Economic context | 7 |  |
| Census County Business Patterns (CCBP) | 21 | 1. Social context | 1 |  |
|  |  | 4. Physical infrastructure | 18 |  |
| Centers for Medicare and Medicaid (CMS) Provider of Services (POS) File | 71 | 5. Healthcare context | 69 |  |
| Common Core of Data (CCD) | 9 | 3. Education | 9 |  |
| Community Resilience Estimates (CRE) | 7 | 1. Social Context | 7 |  |
| County Adjacency File (CAF) | 14 | 6. Geography | 4 |  |
| County Health Rankings (CHR) | 13 | 1. Social context | 2 |  |
|  |  | 4. Physical infrastructure | 1 |  |
|  |  | 5. Healthcare context | 10 |  |
| Dartmouth Atlas of Health Care (DARTMOUTH) | 8 | 5. Healthcare context | 8 |  |
| Environmental Protection Agency (EPAA) | 14 | 4. Physical infrastructure | 4 |  |
| HRSA Medically Underserved Areas (MUA) | 1 | 5. Healthcare context | 1 | 1 |
| Home Health Compare (HHC) | 6 | 5. Healthcare context | 1 |  |
| Local Area Unemployment Statistics (LAUS) | 6 | 2. Economic context | 4 |  |
| Long-term Care: Facts on Care in the U.S. Public Use Data (LTCFOCUS) | 16 | 5. Healthcare context | 16 |  |
| Mapping Medicare Disparities Tool (MMD) | 48 | 5. Healthcare context | 48 |  |
| Medicare Advantage State/County Penetration Files (MP) | 3 | 5. Healthcare context | 3 |  |
| Medicare Geographic Variation Public Use File (MGV) | 16 | 5. Healthcare context | 16 |  |
| National Center for HIV, Viral Hepatitis, STD, and TB Prevention AtlasPlus (CDCAP) | 15 | 5. Healthcare context | 12 |  |
| National Center for Health Statistics Urban-Rural Classification Scheme (NCHS) | 2 | 6. Geography | 2 |  |
| National Environmental Public Health Tracking Network (NEPHTN) | 15 | 4. Physical infrastructure | 8 |  |
| National Oceanic and Atmospheric Administration Climate (NOAAC) | 62 | 4. Physical infrastructure | 61 |  |
| Nursing Home Compare (NHC) | 5 | 5. Healthcare context | 5 |  |
| Physician Compare (PC) | 2 | 5. Healthcare context | 2 |  |
| U.S. Census Bureau, TIGERweb and COVID-19 Demographic and Economic Resources (Census) | 3 | 6. Geography | 3 | 1 |
| Washington University Regional Estimates of Chemical Composition of Fine Particulate Matter using a Combined Geoscience-Statistical Method with Information from Satellites, Models, and Monitors (WUSTL) | 1 | 4. Physical infrastructure | 1 |  |
| amfAR Opioid & Health Indicators Database (AMFAR) | 26 | 5. Healthcare context | 26 |  |
|  | contextual SDoH variables |  | After preprocessing | Final analytic contextual SDoH predictors |
| total | 1033 |  | 961 | 968 |

* All data sources are spatially scaled at the county level.

* All contextual SDoH variables were spatially scaled at the county level and were used as area-level proxies for patients’ social and environmental context, not as individual-level patient attributes.

* The original contextual SDoH inventory included 1,033 county-level variables from public data sources. During preprocessing, variables with high missingness and insufficient variability were excluded, resulting in 961 retained variables. This count included original categorical variables; seven additional dummy variables were generated and added to the analytic dataset. The final modeling-ready contextual SDoH feature set included 968 predictors.

**Table S4.** Performance metrics for XGBoost and Logistic

| \|  \|  \|  \| F1-Score \| C Statistic \| Recall \| Precision \| Specificity \| \| --- \| --- \| --- \| --- \| --- \| --- \| --- \| --- \| \| XGBoost \| full variable \|  \|  \|  \|  \|  \|  \| \| HFpEF \| mean \| 0.370 \| 0.550 \| 0.463 \| 0.321 \| 0.604 \| \|  \| std.dev \| 0.056 \| 0.045 \| 0.136 \| 0.048 \| 0.139 \| \| HFrEF \| mean \| 0.427 \| 0.596 \| 0.481 \| 0.388 \| 0.671 \| \|  \| std.dev \| 0.029 \| 0.024 \| 0.061 \| 0.026 \| 0.058 \| \| Contextual SDoH \|  \|  \|  \|  \|  \|  \| \| HFpEF \| mean \| 0.380 \| 0.528 \| 0.506 \| 0.307 \| 0.550 \| \|  \| std.dev \| 0.040 \| 0.037 \| 0.085 \| 0.027 \| 0.064 \| \| HFrEF \| mean \| 0.331 \| 0.493 \| 0.381 \| 0.300 \| 0.613 \| \|  \| std.dev \| 0.03 \| 0.024 \| 0.086 \| 0.023 \| 0.094 \| \| Clinical \|  \|  \|  \|  \|  \|  \| \| HFpEF \| mean \| 0.364 \| 0.551 \| 0.433 \| 0.322 \| 0.635 \| \|  \| std.dev \| 0.051 \| 0.048 \| 0.104 \| 0.044 \| 0.101 \| \| HFrEF \| mean \| 0.451 \| 0.62 \| 0.536 \| 0.391 \| 0.638 \| \|  \| std.dev \| 0.030 \| 0.030 \| 0.055 \| 0.027 \| 0.048 \| \| Individual SDoH \|  \|  \|  \|  \|  \|  \| \| HFpEF \| mean \| 0.396 \| 0.552 \| 0.553 \| 0.319 \| 0.52 \| \|  \| std.dev \| 0.052 \| 0.045 \| 0.162 \| 0.036 \| 0.171 \| \| HFrEF \| mean \| 0.414 \| 0.562 \| 0.53 \| 0.341 \| 0.556 \| \|  \| std.dev \| 0.025 \| 0.021 \| 0.068 \| 0.018 \| 0.063 \| \| Individual SDoH + Contextual SDoH \|  \|  \|  \|  \|  \|  \| \| HFpEF \| mean \| 0.369 \| 0.551 \| 0.439 \| 0.323 \| 0.63 \| \|  \| std.dev \| 0.048 \| 0.036 \| 0.088 \| 0.034 \| 0.063 \| \| HFrEF \| mean \| 0.375 \| 0.524 \| 0.458 \| 0.323 \| 0.584 \| \|  \| std.dev \| 0.037 \| 0.024 \| 0.097 \| 0.024 \| 0.103 \| \| Clinical+ Individual SDoH \|  \|  \|  \|  \|  \|  \| \| HFpEF \| mean \| 0.330 \| 0.563 \| 0.334 \| 0.340 \| 0.743 \| \|  \| std.dev \| 0.069 \| 0.046 \| 0.100 \| 0.056 \| 0.072 \| \| HFrEF \| mean \| 0.454 \| 0.629 \| 0.524 \| 0.402 \| 0.662 \| \|  \| std.dev \| 0.031 \| 0.026 \| 0.057 \| 0.028 \| 0.047 \| \| LRC \| full variable \|  \|  \|  \|  \|  \|  \| \| HFpEF \| mean \| 0.416 \| 0.603 \| 0.494 \| 0.360 \| 0.654 \| \|  \| std.dev \| 0.045 \| 0.040 \| 0.067 \| 0.038 \| 0.040 \| \| HFrEF \| mean \| 0.470 \| 0.640 \| 0.558 \| 0.407 \| 0.649 \| \|  \| std.dev \| 0.021 \| 0.019 \| 0.033 \| 0.018 \| 0.022 \| \| Contextual SDoH \|  \|  \|  \|  \|  \|  \| \| HFpEF \| mean \| 0.382 \| 0.541 \| 0.501 \| 0.310 \| 0.561 \| \|  \| std.dev \| 0.044 \| 0.044 \| 0.078 \| 0.033 \| 0.055 \| \| HFrEF \| mean \| 0.236 \| 0.500 \| 0.510 \| 0.153 \| 0.490 \| \|  \| std.dev \| 0.232 \| 0.000 \| 0.502 \| 0.151 \| 0.502 \| \| Clinical \|  \|  \|  \|  \|  \|  \| \| HFpEF \| mean \| 0.396 \| 0.586 \| 0.475 \| 0.342 \| 0.640 \| \|  \| std.dev \| 0.051 \| 0.041 \| 0.080 \| 0.040 \| 0.043 \| \| HFrEF \| mean \| 0.461 \| 0.637 \| 0.534 \| 0.406 \| 0.662 \| \|  \| std.dev \| 0.021 \| 0.018 \| 0.032 \| 0.019 \| 0.023 \| \| Individual SDoH \|  \|  \|  \|  \|  \|  \| \| HFpEF \| mean \| 0.395 \| 0.573 \| 0.506 \| 0.326 \| 0.586 \| \|  \| std.dev \| 0.045 \| 0.044 \| 0.072 \| 0.036 \| 0.049 \| \| HFrEF \| mean \| 0.432 \| 0.585 \| 0.550 \| 0.357 \| 0.572 \| \|  \| std.dev \| 0.021 \| 0.021 \| 0.035 \| 0.017 \| 0.028 \| \| Individual SDoH+ Contextual SDoH \|  \|  \|  \|  \|  \|  \| \| HFpEF \| mean \| 0.393 \| 0.559 \| 0.510 \| 0.322 \| 0.575 \| \|  \| std.dev \| 0.042 \| 0.041 \| 0.076 \| 0.032 \| 0.055 \| \| HFrEF \| mean \| 0.424 \| 0.577 \| 0.527 \| 0.356 \| 0.589 \| \|  \| std.dev \| 0.023 \| 0.021 \| 0.042 \| 0.018 \| 0.033 \| \| Clinical+ Individual SDoH \|  \|  \|  \|  \|  \|  \| \| HFpEF \| mean \| 0.400 \| 0.580 \| 0.491 \| 0.339 \| 0.622 \| \|  \| std.dev \| 0.047 \| 0.043 \| 0.078 \| 0.037 \| 0.049 \| \| HFrEF \| mean \| 0.474 \| 0.648 \| 0.563 \| 0.409 \| 0.650 \| \|  \| std.dev \| 0.020 \| 0.019 \| 0.031 \| 0.018 \| 0.024 \| |
| --- | --- | --- | --- | --- | --- | --- | --- | --- | --- | --- | --- | --- | --- | --- | --- | --- | --- | --- | --- | --- | --- | --- | --- | --- | --- | --- | --- | --- | --- | --- | --- | --- | --- | --- | --- | --- | --- | --- | --- | --- | --- | --- | --- | --- | --- | --- | --- | --- | --- | --- | --- | --- | --- | --- | --- | --- | --- | --- | --- | --- | --- | --- | --- | --- | --- | --- | --- | --- | --- | --- | --- | --- | --- | --- | --- | --- | --- | --- | --- | --- | --- | --- | --- | --- | --- | --- | --- | --- | --- | --- | --- | --- | --- | --- | --- | --- | --- | --- | --- | --- | --- | --- | --- | --- | --- | --- | --- | --- | --- | --- | --- | --- | --- | --- | --- | --- | --- | --- | --- | --- | --- | --- | --- | --- | --- | --- | --- | --- | --- | --- | --- | --- | --- | --- | --- | --- | --- | --- | --- | --- | --- | --- | --- | --- | --- | --- | --- | --- | --- | --- | --- | --- | --- | --- | --- | --- | --- | --- | --- | --- | --- | --- | --- | --- | --- | --- | --- | --- | --- | --- | --- | --- | --- | --- | --- | --- | --- | --- | --- | --- | --- | --- | --- | --- | --- | --- | --- | --- | --- | --- | --- | --- | --- | --- | --- | --- | --- | --- | --- | --- | --- | --- | --- | --- | --- | --- | --- | --- | --- | --- | --- | --- | --- | --- | --- | --- | --- | --- | --- | --- | --- | --- | --- | --- | --- | --- | --- | --- | --- | --- | --- | --- | --- | --- | --- | --- | --- | --- | --- | --- | --- | --- | --- | --- | --- | --- | --- | --- | --- | --- | --- | --- | --- | --- | --- | --- | --- | --- | --- | --- | --- | --- | --- | --- | --- | --- | --- | --- | --- | --- | --- | --- | --- | --- | --- | --- | --- | --- | --- | --- | --- | --- | --- | --- | --- | --- | --- | --- | --- | --- | --- | --- | --- | --- | --- | --- | --- | --- | --- | --- | --- | --- | --- | --- | --- | --- | --- | --- | --- | --- | --- | --- | --- | --- | --- | --- | --- | --- | --- | --- | --- | --- | --- | --- | --- | --- | --- | --- | --- | --- | --- | --- | --- | --- | --- | --- | --- | --- | --- | --- | --- | --- | --- | --- | --- | --- | --- | --- | --- | --- | --- | --- | --- | --- | --- | --- | --- | --- | --- | --- | --- | --- | --- | --- | --- | --- | --- | --- | --- | --- | --- | --- | --- | --- | --- | --- | --- | --- | --- | --- | --- | --- | --- | --- | --- | --- | --- | --- | --- | --- | --- | --- | --- | --- | --- | --- | --- | --- | --- | --- | --- | --- | --- | --- | --- | --- | --- | --- | --- | --- | --- | --- | --- | --- | --- | --- | --- | --- | --- | --- | --- | --- | --- | --- | --- | --- | --- | --- | --- | --- |

**Table S5.** Opportunity of equality measured by false negative rate by different models on various feature sets

| Attributed feature | HFpEF | HFrEF |
| --- | --- | --- |
| Hispanic white | 1.230 | 0.988 |
| Sex | 1.281 | 1.030 |
| Black and White | 0.872 | 0.920 |

**Figure S1.** Workflow to identify cohort and build up ML model.


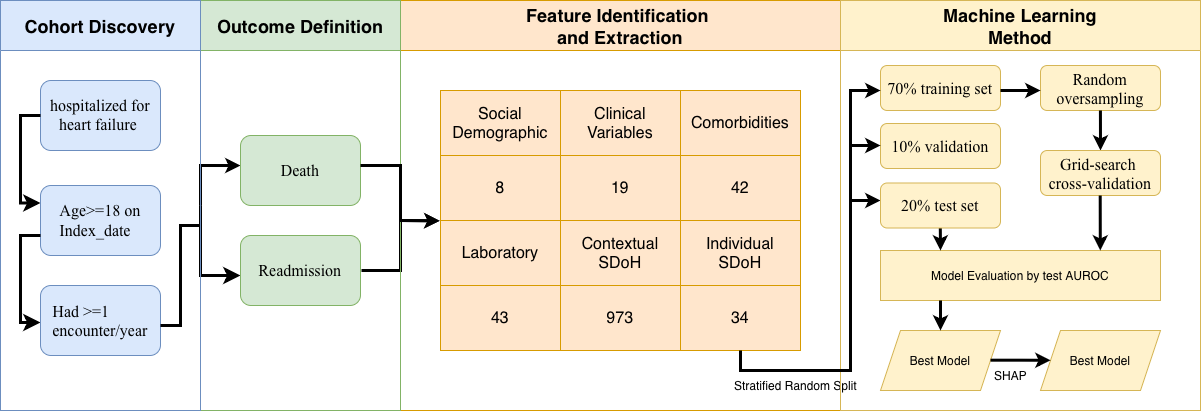


**Figure S2.** 6-month readmission/death risk by machine learning prediction risk decile using XGboost model. (a) HFpEF population (b) HFrEF population

**(a)
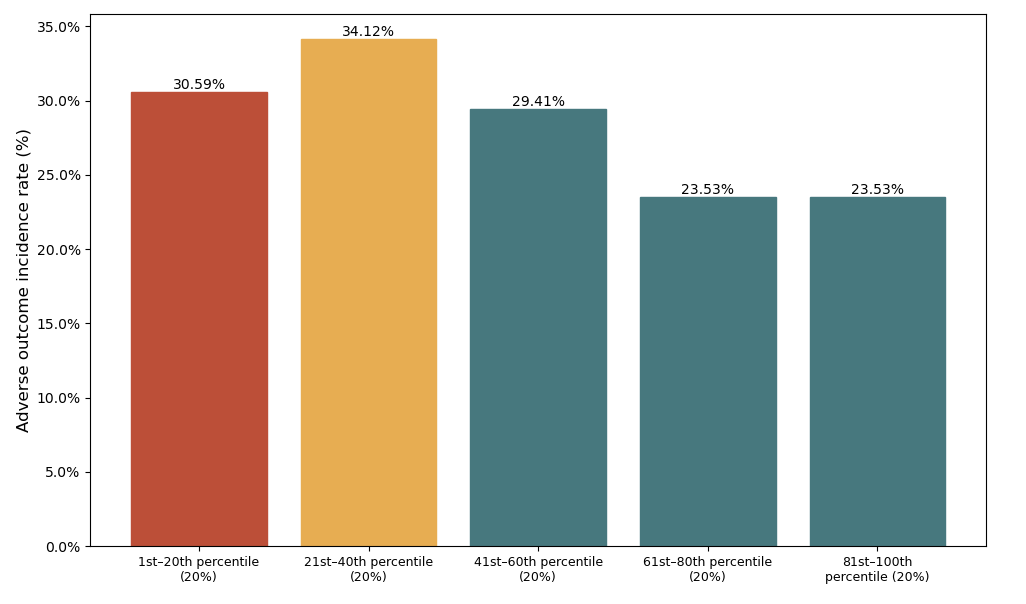
**

**(b)
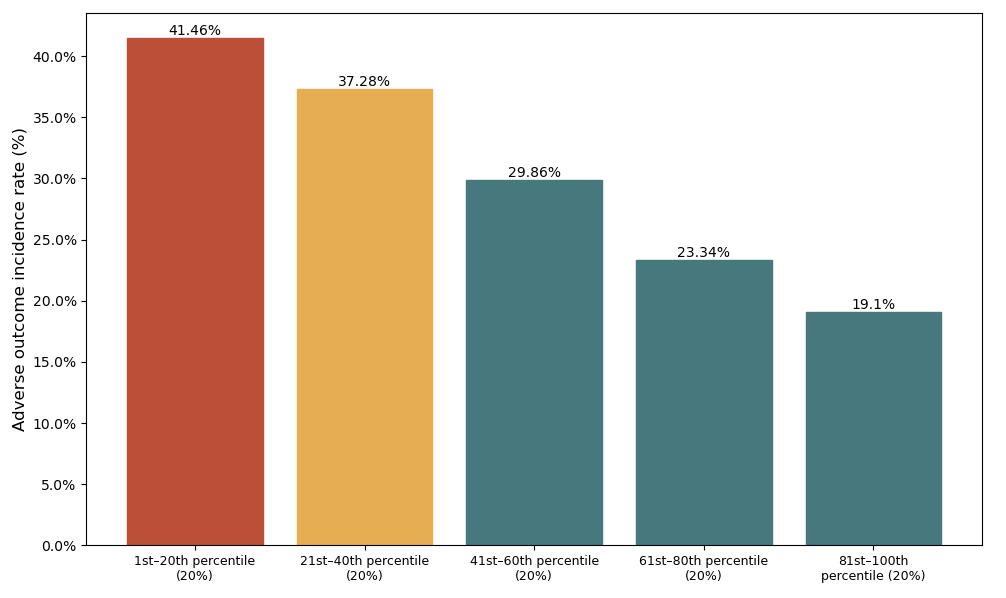
**

Figure S3. SHAP values of important predictions from the original XGboost. (a) HFpEF (b) HFrEF.

(a)
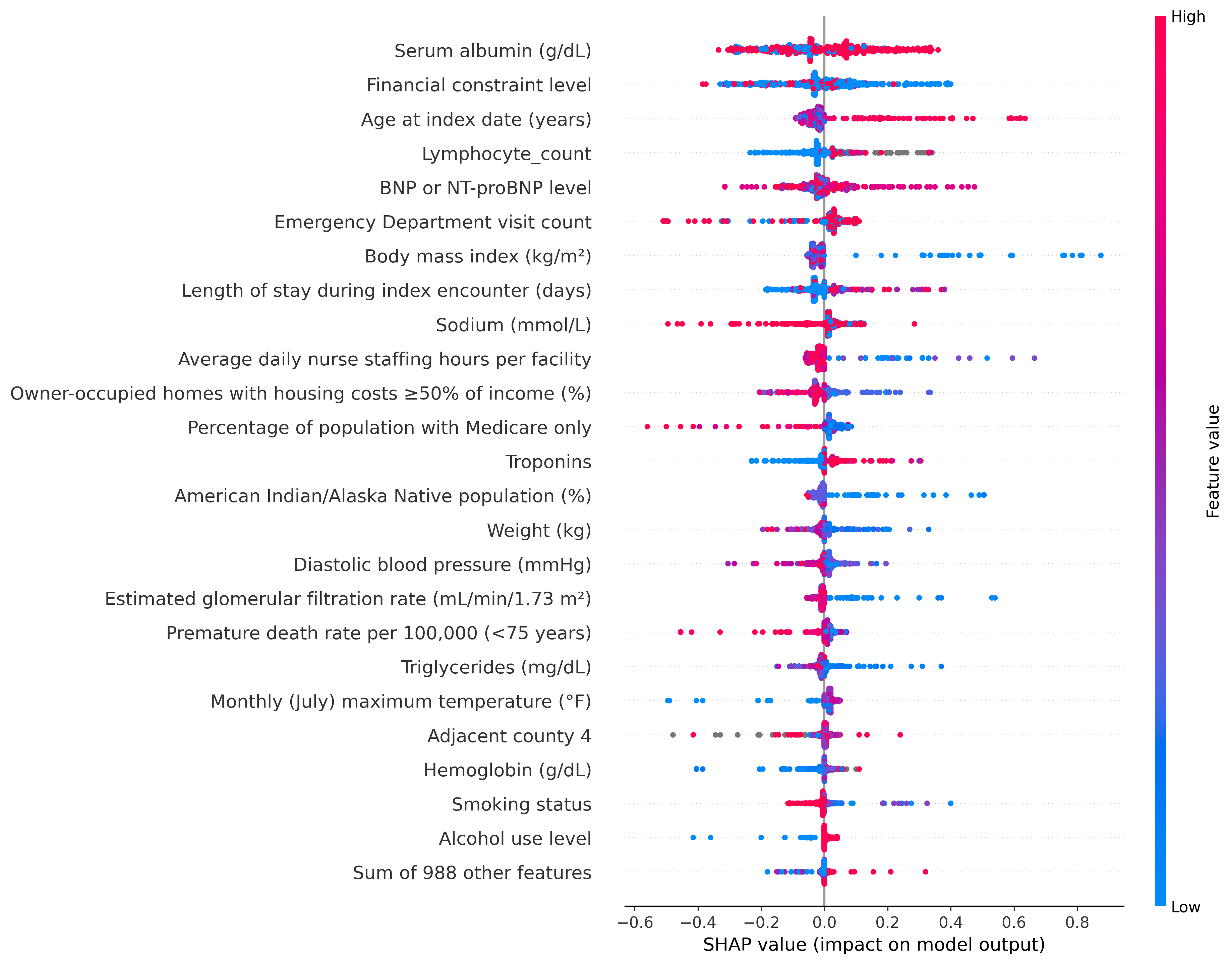

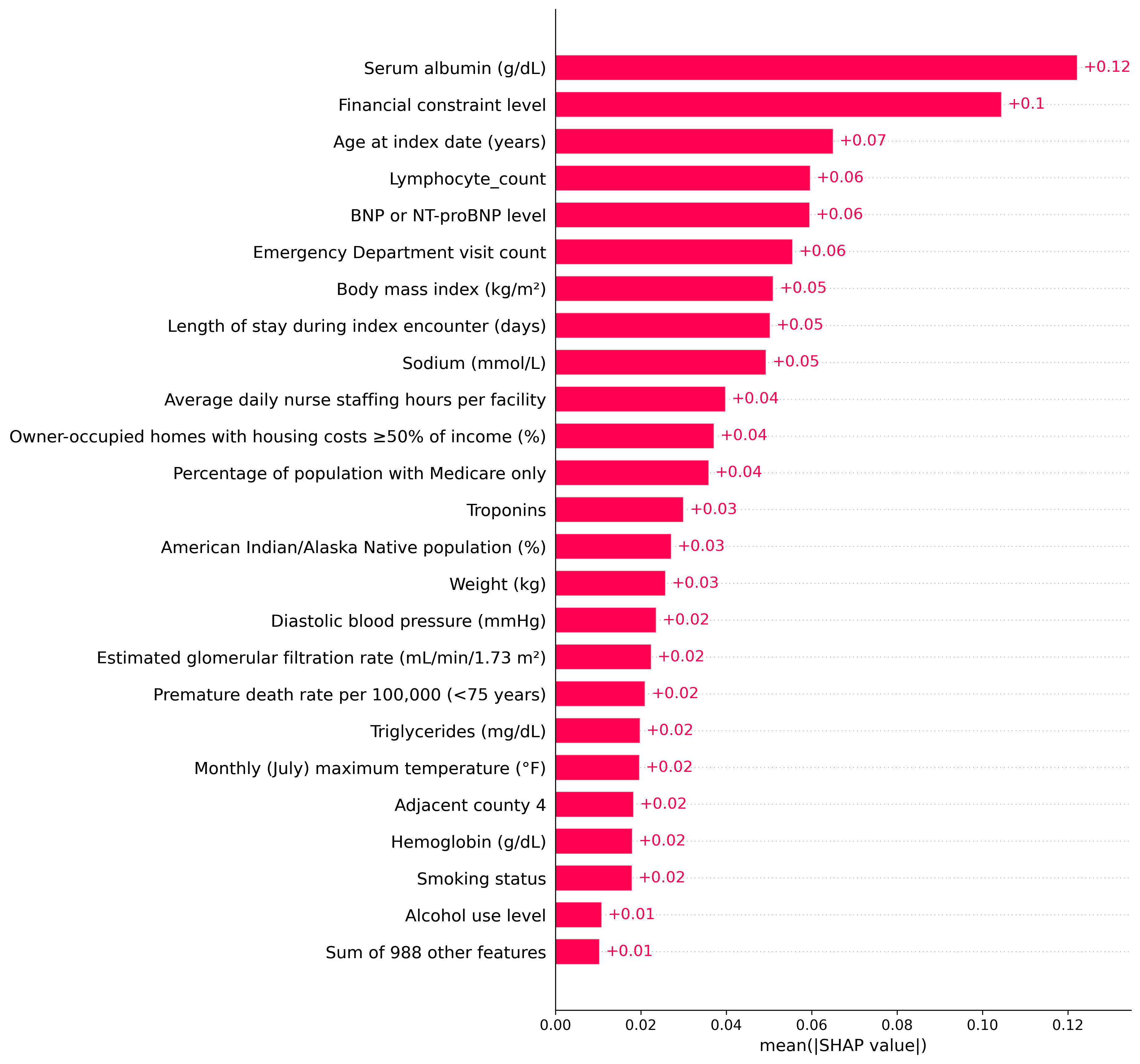


(b)
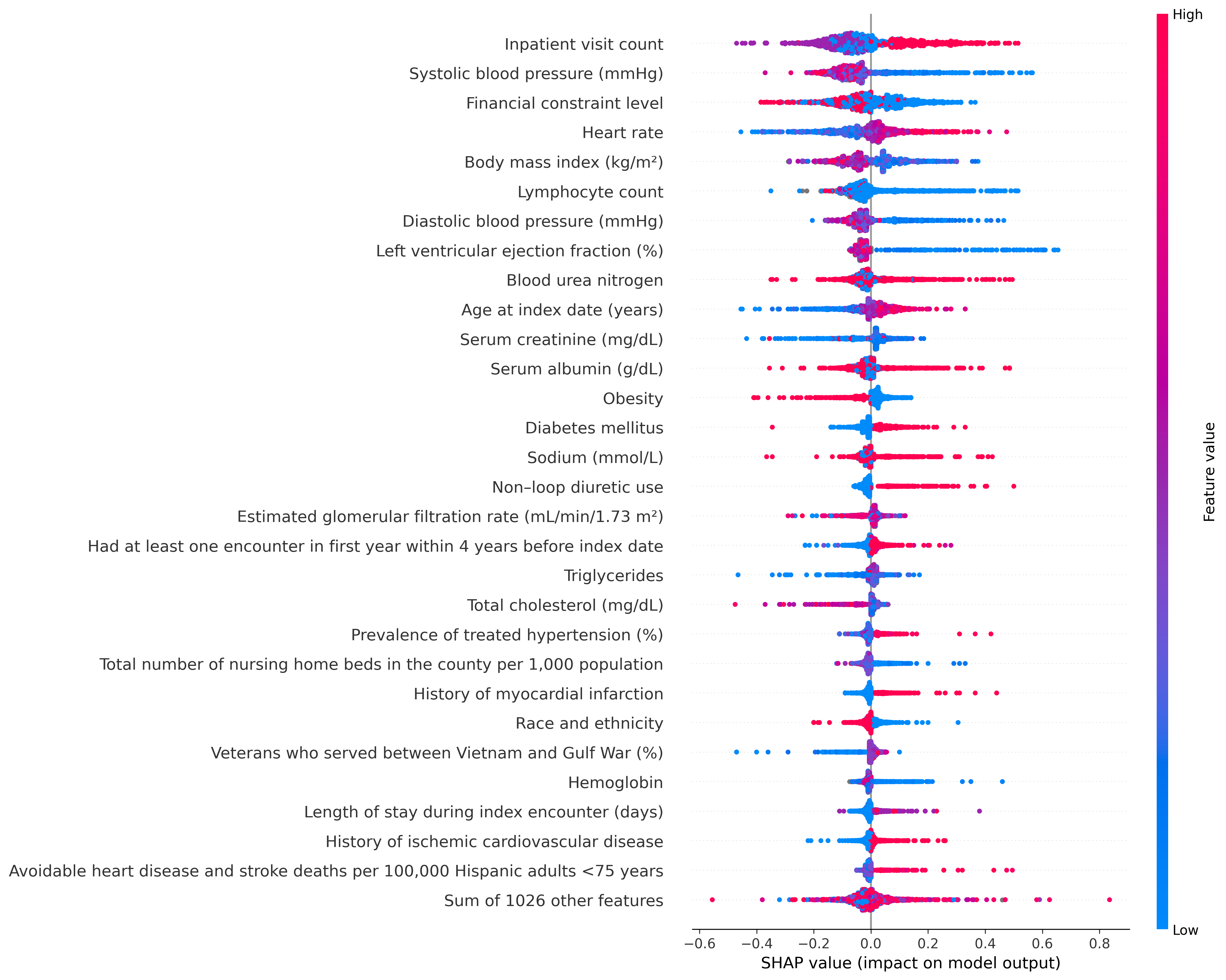

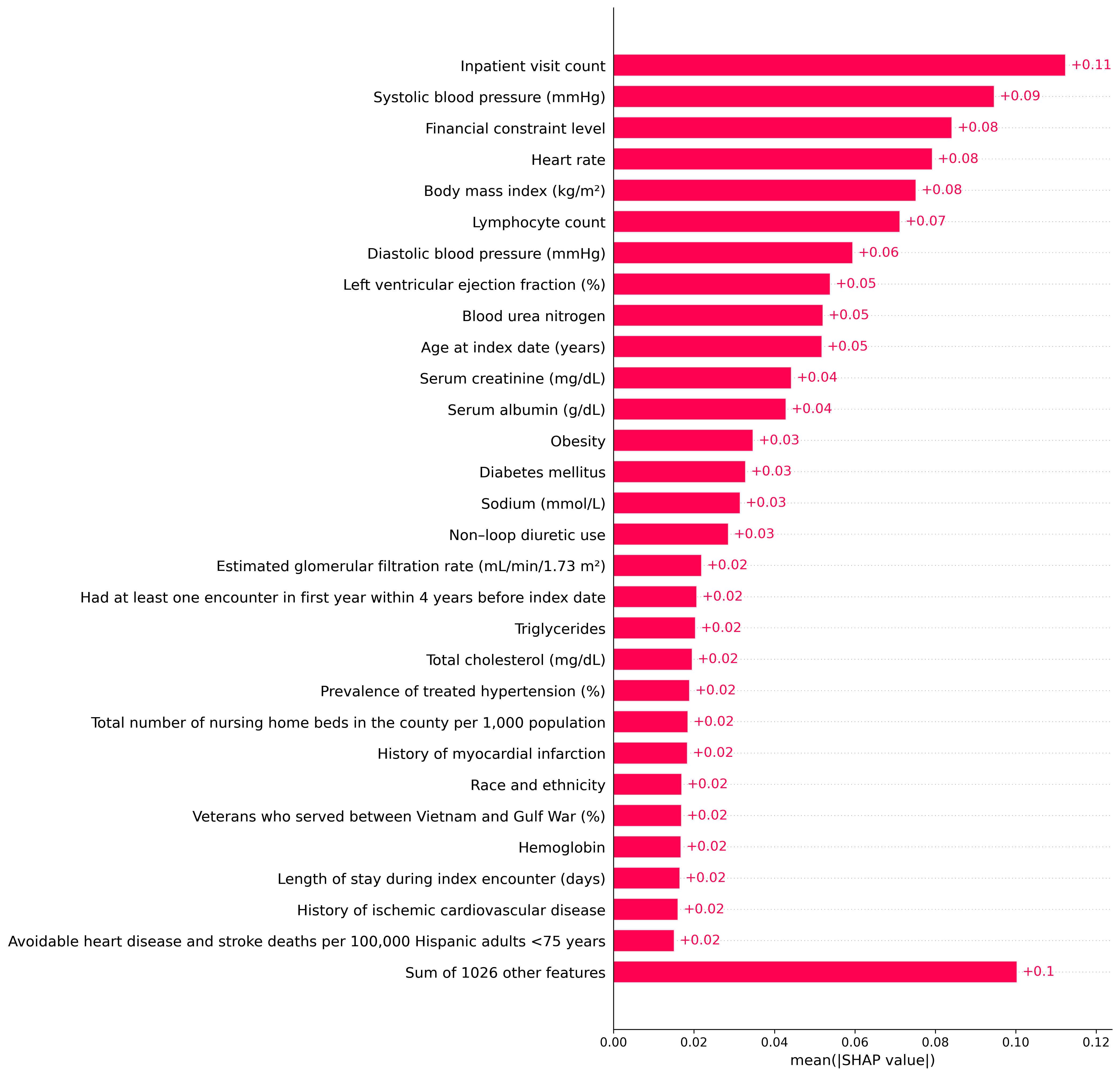


**Figure S4.** The causal discovery results on full data with XGBoost model from SHAP analysis. These three images are results from CPC models. The blue nodes present SDoH and demographics variables, the green nodes stand for comorbidities and medication variables, purple nodes stand for the clinical variables, and the red node indicates the outcome. The red edges represent the indirect relationships between SDoH and outcome. (a) HFpEF (b) HFrEF


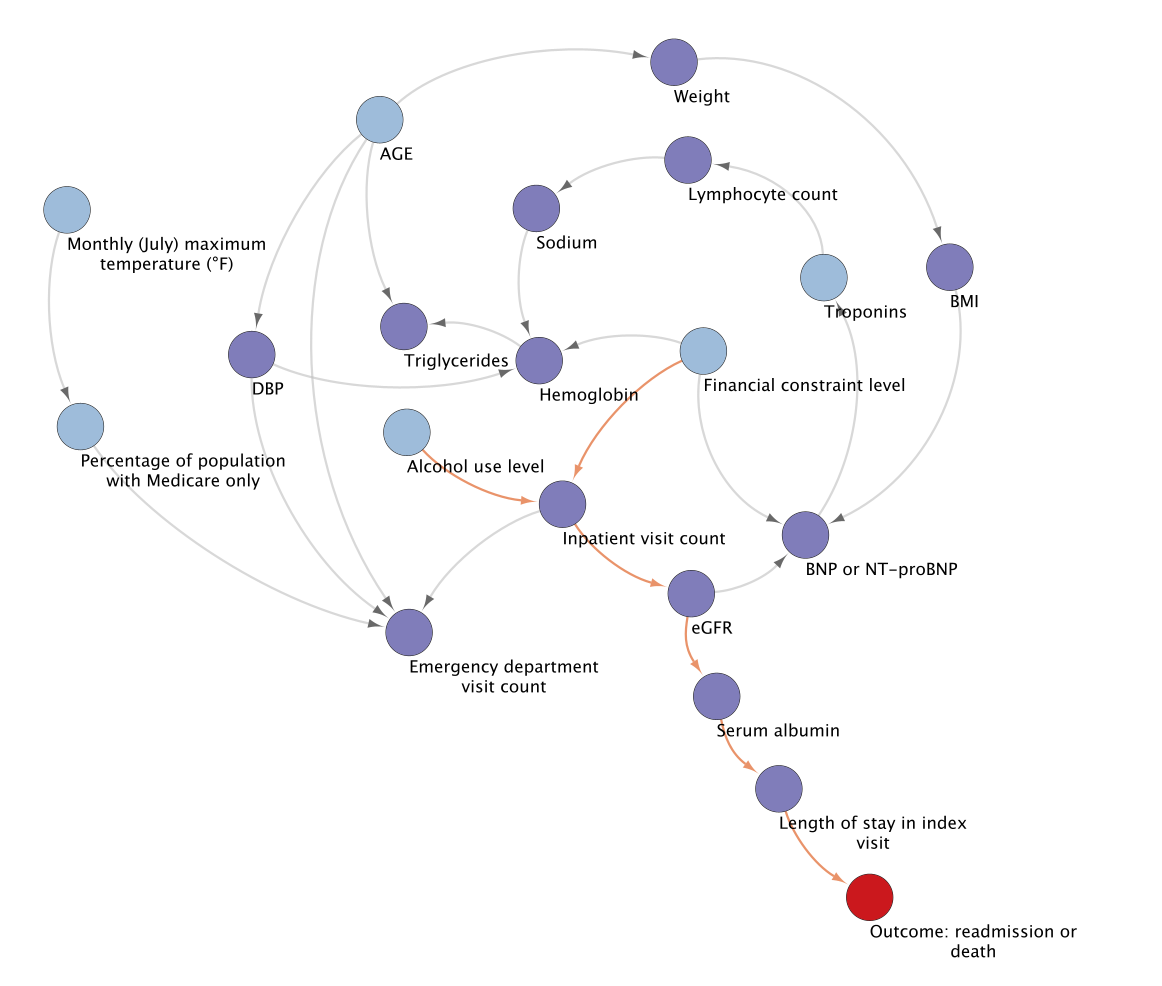
**(a)**

**
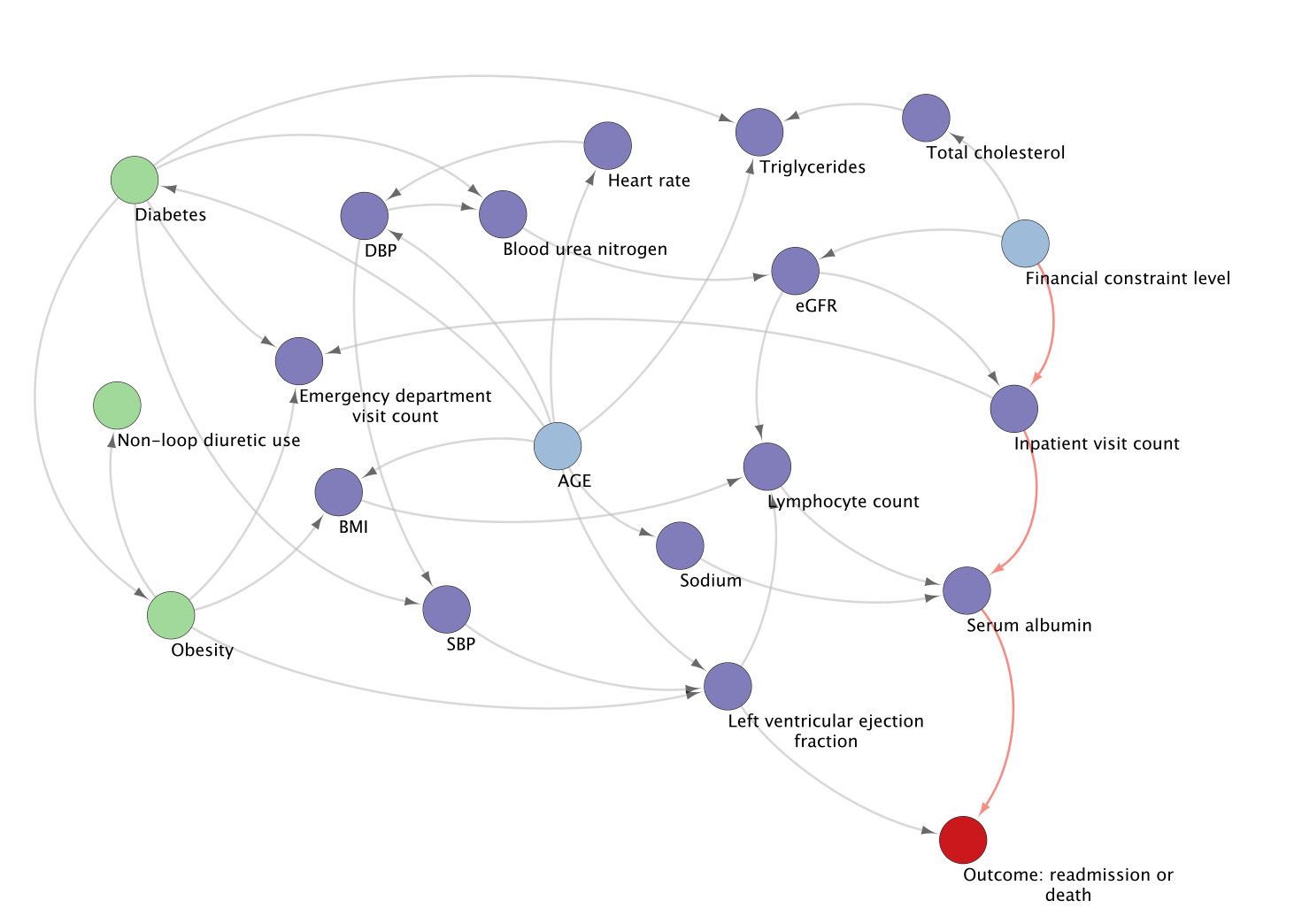
(b)**

**Figure S5.** Evaluation of algorithm fairness with a focus on False negative rate disparity with XGBoost. By (a) sex, (b) non-Hispanic white, non-Hispanic black and Hispanic

**(a)** **
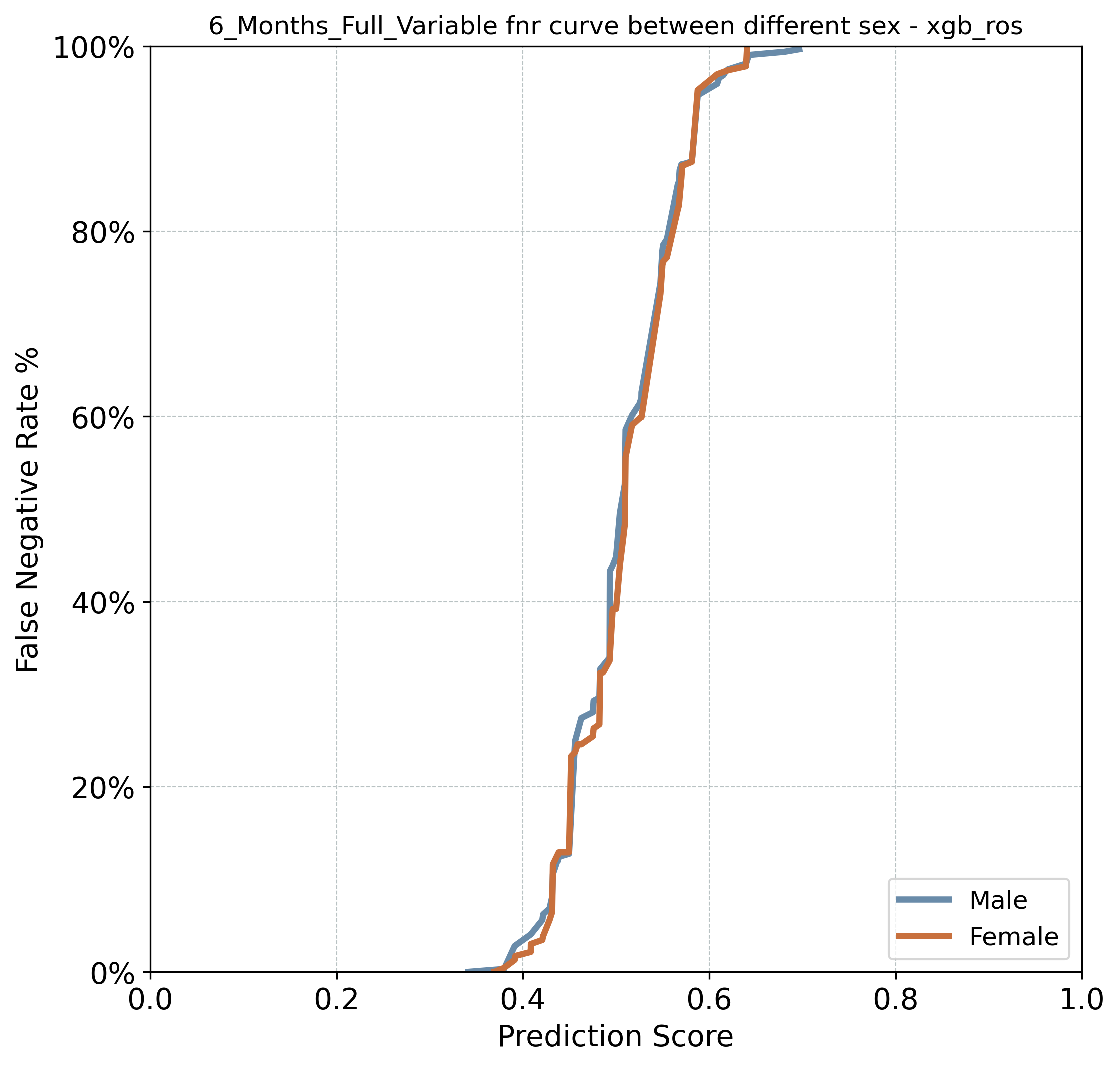
(b)**
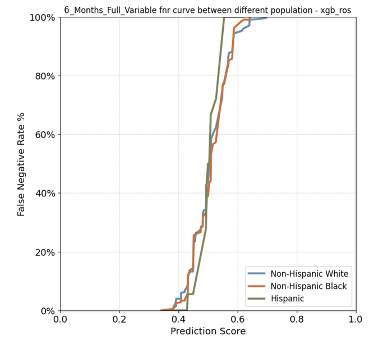


Figure S6. Impact of bias mitigation on Model Performance. The x-axis shows **Equality of Opportunity**, and the y-axis shows **C statistic**. Mitigation models reduced bias but also lowered C statistic (a) **FNR Ratio: Black/White** in HFpEF (b) **FNR Ratio: Hispanic/White** in HFrEF population.

**(a)**
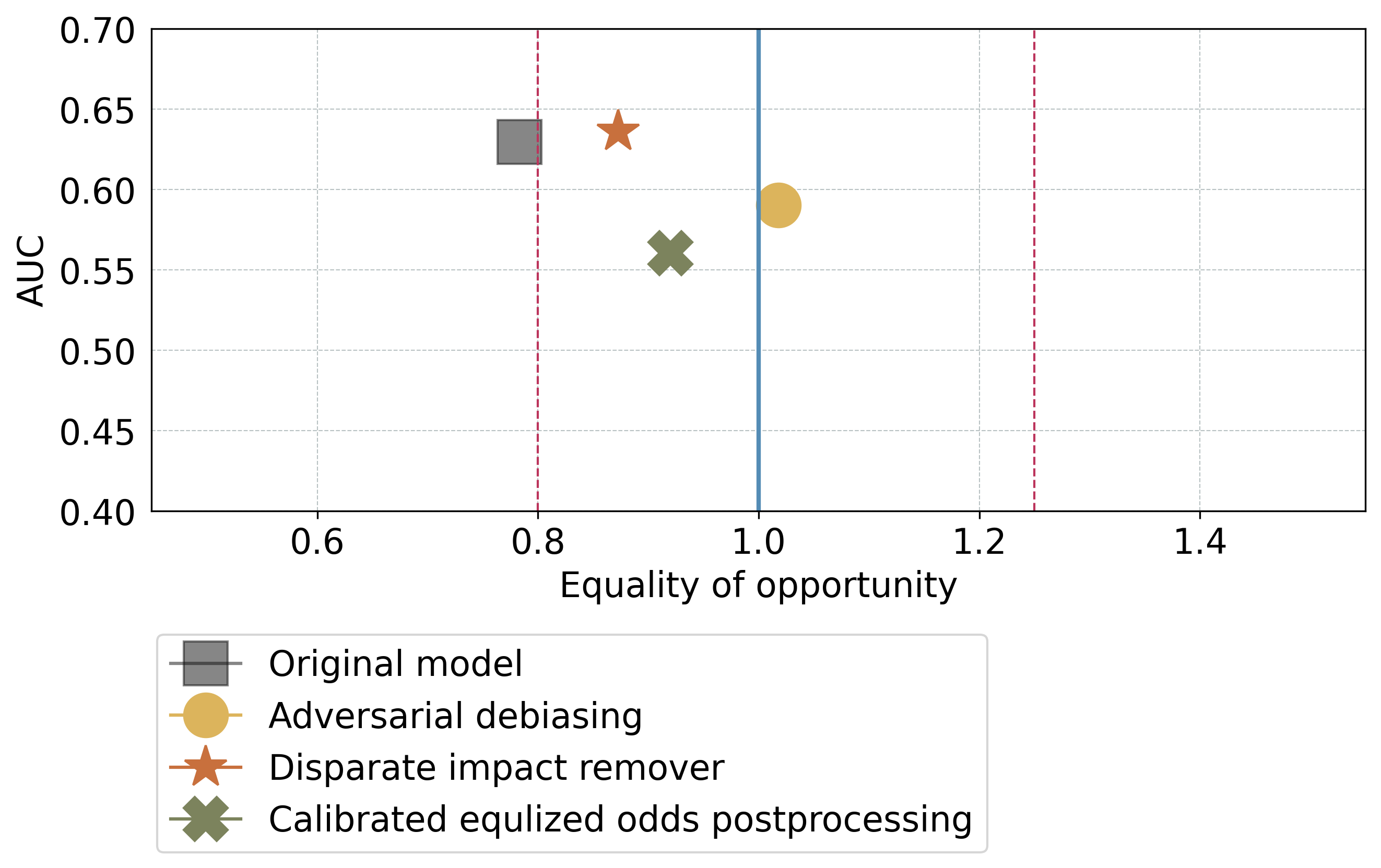


**(b)**
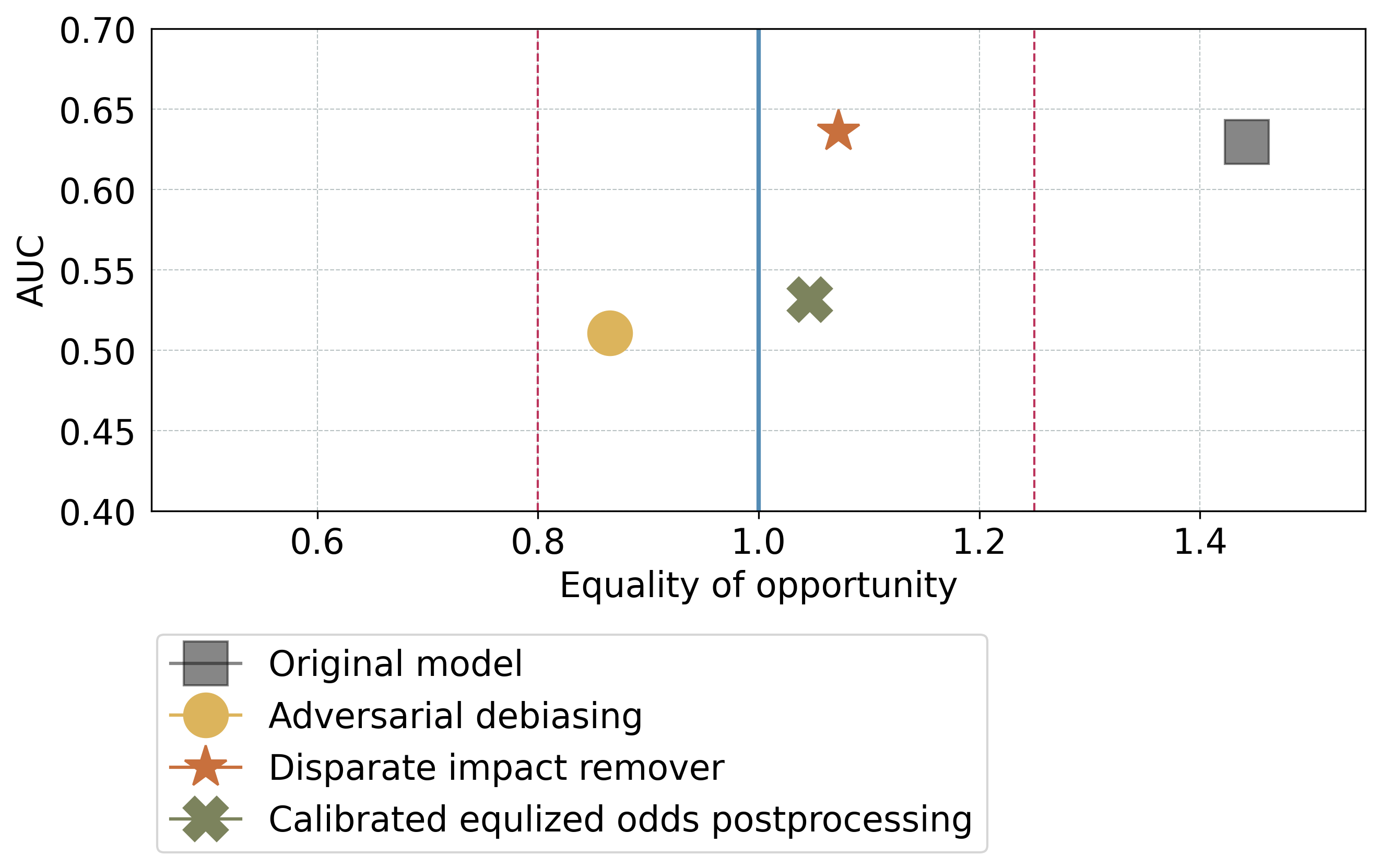

Supplement: ooag136_Supplementary_Data [file ooag136_supplementary_data.docx]
